# A systematic review of empirical studies examining mechanisms of implementation in health

**DOI:** 10.1186/s13012-020-00983-3

**Published:** 2020-04-16

**Authors:** Cara C. Lewis, Meredith R. Boyd, Callie Walsh-Bailey, Aaron R. Lyon, Rinad Beidas, Brian Mittman, Gregory A. Aarons, Bryan J. Weiner, David A. Chambers

**Affiliations:** 1 Kaiser Permanente Washington Health Research Institute, 1730 Minor Avenue, Suite 1600, Seattle, WA 98101 USA; 2 Department of Psychological and Brain Sciences, Indiana University, 1101 E 10th Street, Bloomington, IN 47405 USA; 3 Department of Psychiatry and Behavioral Sciences, School of Medicine, University of Washington, 1959 NE Pacific Avenue, Seattle, WA 98195 USA; 4 Department of Psychology, University of California Los Angeles, 1177 Franz Hall, 502 Portola Plaza, Los Angeles, CA 90095 USA; 5 Brown School, Washington University in St. Louis, 1 Brookings Drive, St. Louis, MO 63130 USA; 6 Department of Psychiatry, Perelman School of Medicine, University of Pennsylvania, 3535 Market Street, Philadelphia, PA 19104 USA; 7 Department of Research and Evaluation, Kaiser Permanente Southern California, 100 S Los Robles Avenue, Pasadena, CA 91101 USA; 8 Department of Psychiatry, School of Medicine, University of California San Diego, 9500 Gilman Drive, La Jolla, CA 92093 USA; 9 Department of Health Services, University of Washington, 1959 NE Pacific Street, Seattle, WA 98195 USA; 10 Division of Cancer Control and Population Science, National Cancer Institute, 9609 Medical Center Drive, Rockville, MD 20850 USA

**Keywords:** Mechanism, Moderator, Mediator, Determinant, Implementation, Causal model, Theory

## Abstract

**Background:**

Understanding the mechanisms of implementation strategies (i.e., the processes by which strategies produce desired effects) is important for research to understand why a strategy did or did not achieve its intended effect, and it is important for practice to ensure strategies are designed and selected to directly target determinants or barriers. This study is a systematic review to characterize how mechanisms are conceptualized and measured, how they are studied and evaluated, and how much evidence exists for specific mechanisms.

**Methods:**

We systematically searched PubMed and CINAHL Plus for implementation studies published between January 1990 and August 2018 that included the terms “mechanism,” “mediator,” or “moderator.” Two authors independently reviewed title and abstracts and then full texts for fit with our inclusion criteria of empirical studies of implementation in health care contexts. Authors extracted data regarding general study information, methods, results, and study design and mechanisms-specific information. Authors used the Mixed Methods Appraisal Tool to assess study quality.

**Results:**

Search strategies produced 2277 articles, of which 183 were included for full text review. From these we included for data extraction 39 articles plus an additional seven articles were hand-entered from only other review of implementation mechanisms (total = 46 included articles). Most included studies employed quantitative methods (73.9%), while 10.9% were qualitative and 15.2% were mixed methods. Nine unique versions of models testing mechanisms emerged. Fifty-three percent of the studies met half or fewer of the quality indicators. The majority of studies (84.8%) only met three or fewer of the seven criteria stipulated for establishing mechanisms.

**Conclusions:**

Researchers have undertaken a multitude of approaches to pursue mechanistic implementation research, but our review revealed substantive conceptual, methodological, and measurement issues that must be addressed in order to advance this critical research agenda. To move the field forward, there is need for greater precision to achieve conceptual clarity, attempts to generate testable hypotheses about how and why variables are related, and use of concrete behavioral indicators of proximal outcomes in the case of quantitative research and more directed inquiry in the case of qualitative research.

Contributions to the literature statement

- This is the first systematic review of implementation mechanisms across health that assesses the quality of studies and the extent to which they offer evidence in support of establishing mechanisms of implementation.
- We summarize nine examples of models for evaluating mechanisms.
- We offer conceptual, theoretical, and methodological guidance for the field to contribute to the study of implementation mechanisms.

## Background

Implementation research is the scientific evaluation of strategies or methods used to support the integration of evidence-based practices or programs (EBPs) into healthcare settings to enhance the quality and effectiveness of services [1]. There is mounting evidence that multi-faceted or blended implementation strategies are necessary (i.e., a discrete strategy is insufficient) [2, 3], but we have a poor understanding of how and why these strategies work. Mechanistic research in implementation science is in an early phase of development. As of 2016, there were only nine studies included in one systematic review of implementation *mediators*^1^ specific to the field of mental health. Mediators are an intervening variable that may statistically account for the relation between an implementation strategy and outcome. We define the term *mechanism* as a process or event through which an implementation strategy operates to affect one or more implementation outcomes (see Table 1 for key terms and definitions used throughout this manuscript). Mechanisms offer causal pathways explaining how strategies operate to achieve desired outcomes, like changes in care delivery. Some researchers conflate moderators, mediators, and mechanisms [6], using the terms interchangeably [7]. Mediators and moderators can point toward mechanisms, but they are not all mechanisms as they typically are insufficient to explain exactly how change came about.

**Table 1:** Terms and definitions

Term	Definition
Mechanism	Process or event through which an implementation strategy operates to affect desired implementation outcomes.
Precondition	Factor that is necessary in order for an implementation mechanism to be activated.
Strategy	Methods used to promote the implementation of an evidence-based practice or program
Determinant	Also commonly referred to as “barriers” and “facilitators,” a factor that enables or hinders the implementation strategy from eliciting the desired effect.
Mediator	Intervening variable that may account for the relationship between the implementation strategy and the implementation outcome.
Moderator	Factor that increase or decrease the level of influence of an implementation strategy.
Proximal outcome	The product of the implementation strategy that is realized because of its specific mechanism of action, the most immediate, observable outcome in the causal pathway.
Distal outcome	Outcome that the implementation processes is ultimately intended to achieve, not the most immediate outcome in the causal pathway.

In addition to these linguistic inconsistencies and lack of conceptual clarity, there is little attention paid to the criteria for establishing a mechanistic relation. Originally, Bradford-Hill [8], and more recently Kazdin offers [4] at least seven criteria for establishing mechanisms of psychosocial treatments that are equally relevant to implementation strategies: strong association, specificity, consistency, experimental manipulation, timeline, gradient, plausibility, or coherence (see Table 2 for definitions). Taken together, these criteria can guide study designs for building the case for mechanisms over time. In lieu of such criteria, disparate models and approaches for investigating mechanisms are likely to exist that make synthesizing findings across studies quite challenging. Consequently, the assumption that more strategies will achieve better results is likely to remain, driving costly and imprecise approaches to implementation.

**Table 2:** Kazdin’s criteria for establishing a mechanism

Term	Definition
Strong association	Association between implementation strategy and mechanism AND between mechanism and behavior change.
Specificity	One plausible construct accounts for behavior change.
Consistency	Replication of observed results across studies, samples, and conditions.
Experimental manipulation	Direct manipulation of implementation strategy or proposed mediator or mechanism shows impact on outcomes.
Timeline	Causes and mediators temporally precede effects and outcomes.
Gradient	Dose response relationship between mediator and outcome.
Plausibility or coherence	Explanation invokes other info and steps in a process-outcome relation that are reasonable or supported by other research.

Understanding the mechanisms of implementation strategies, defined as the processes by which strategies produce desired effects [4, 8], is important for both research and practice. For research, it is important to specify and examine mechanisms of implementation strategies, especially in the case of null studies, in order to understand why a strategy did or did not achieve its intended effect. For practice, it is crucial to understand mechanisms so that strategies are designed and selected to directly target implementation determinants or barriers. In the absence of this kind of intentional, a priori matching (i.e., strategy targets determinant), it is possible that the “wrong” (or perhaps less potent) strategy will be deployed. This phenomenon of mismatched strategies and determinants was quite prevalent among the 22 tailored improvement intervention studies included in Bosch et al.’s [9] multiple case study analysis. Upon examining the timing of determinant identification and the degree to which included studies informed tailoring of the type versus the content of the strategies using determinant information, they discovered frequent determinant-strategy mismatch across levels of analysis (e.g., clinician-level strategies were used to address barriers that were at the organizational level) [9]. Perhaps what is missing is a clear articulation of implementation mechanisms to inform determinant-strategy matching. We argue that, ultimately, knowledge of mechanisms would help to create a more rational, efficient bundle of implementation strategies that fit specific contextual challenges.

Via a systematic review, we sought to understand how mechanisms are conceptualized and measured, how they are studied (by characterizing the wide array of models and designs used to evaluate mechanisms) and evaluated (by applying Kazdin’s seven criteria), and how much evidence exists for specific mechanisms. In doing so, we offer a rich characterization of the current state of the evidence. In reflecting on this evidence, we provide recommendations for future research to optimize their contributions to mechanistic implementation science.

## Methods

### Search protocol

The databases, PubMed and CINAHL Plus, were chosen because of their extensive collection of over 32 million combined citations of medical, nursing and allied health, and life science journals, as well as inclusiveness of international publications. We searched both databases in August 2018 for empirical studies published between January 1990 and August 2018 testing candidate mechanisms of implementation strategies. This starting date was selected given that the concept of evidence-based practice/evidence-based treatment/evidence-based medicine first gained prominence in the 1990’s with the field of implementation science following in response to a growing consciousness of the research to practice gap [10, 11]. The search terms were based on input from all authors who represent a variety of methodological and content expertise related to implementation science and reviewed by a librarian; see Table 3 for all search terms. The search string consisted of three levels with terms reflecting (1) implementation science, (2) evidence-based practice (EBP), and (3) mechanism. We adopted Kazdin’s [4] definition of mechanisms, which he indicates are the basis of an effect. Due to the diversity of definitions that exist in the literature, the term “mechanism” was supplemented with the terms “mediator” and “moderator” to ensure all relevant studies were collected.

**Table 3:** Search strategy

Search terms	Explanation
Implement* OR disseminate* OR “knowledge translation”	These terms were chosen to target Implementation Science literature.
AND	
“empirically supported treatment” OR “evidence-based practice” OR “evidence-based treatment” OR innovation OR guideline	These terms were chosen to target the implementation evidence-based practices
AND	
Mediate* OR moderator OR mechanism*	These terms were chosen to target mechanisms explaining the implementation of evidence-based practices
NOT	
Biology OR microbiology	These terms were chosen to exclude mechanistic studies in biology and microbiology

### Study inclusion and exclusion criteria

Studies were included if they were considered an empirical implementation study (i.e., original data collection) and statistically tested or qualitatively explored mechanisms, mediators, or moderators. We did not include dissemination studies given the likely substantive differences between strategies, mechanisms, and outcomes. Specifically, we align with the distinction made between dissemination and implementation put forth by the National Institutes of Health program announcement for Dissemination and Implementation Research in Health that describes dissemination as involving distribution of evidence to a target audience (i.e., communication of evidence) and implementation as involving use of strategies to integrate evidence into target settings (i.e., use of evidence in practice) [12]. However, the word “dissemination” was included in our search terms because of the tendency of some researchers to use “implementation” and “dissemination” interchangeably. Studies were excluded if they were not an implementation study, used the terms “mediator,” “moderator,” or “mechanism” in a different context (i.e., conflict mediator), did not involve the implementation of an EBP, or were a review, concept paper, or opinion piece rather than original research. All study designs were considered. Only studies in English were assessed. See Additional File 1 for exclusion criteria and definitions. We strategically cast a wide net and limited our exclusions so as to characterize the broad range of empirical studies of implementation mechanisms.

Citations generated from the search of PubMed and CINAHL were loaded into EPPI Reviewer 4, an online software program used for conducting literature reviews [13]. Duplicate citations were identified for removal via the duplicate checking function in EPPI and via manual searching. Two independent reviewers (MRB, CWB) screened the first ten citations on title and abstract for inclusion. They then met to clarify inclusion and exclusion criteria with the authorship team, as well as add additional criteria if necessary, and clarify nuances of the inclusion/exclusion coding system (see Additional File 1 for exclusion criteria and definitions). The reviewers met once a week to compare codes and resolve discrepancies through discussion. If discrepancies could not be easily resolved through discussion among the two reviewers, the first author (CCL) made a final determination. During full text review, additional exclusion coding was applied for criteria that could not be discerned from the abstract; articles were excluded at this phase if they only mentioned the study of mechanisms in the discussion or future directions. Seven studies from the previous systematic review of implementation mechanisms [14] were added to our study for data extraction; these studies likely did not appear in our review due to differences in the search strategy in that the review undertaken by Williams hand searched published reviews of implementation strategies in mental health.

### Study quality assessment

The methodological quality of included studies was assessed using the Mixed Methods Appraisal Tool (MMAT-version 2018) [15]. This tool has been utilized in over three dozen systematic reviews in the health sciences. The MMAT includes two initial screening criteria that assess for the articulation of a clear research question/objective and for the appropriateness of the data collected to address the research question. Studies must receive a “yes” in order to be included. The tool contains a subset of questions to assess for quality for each study type—qualitative, quantitative, and mixed methods. Table 4 summarizes the questions by which studies were evaluated, such as participant recruitment and relevance and quality of measures. Per the established approach to MMAT application, a series of four questions specific to each study design type are assigned a dichotomous “yes” or “no” answer. Studies receive 25 percentage points for each “yes” response. Higher percentages reflect higher quality, with 100% indicating all quality criteria were met. The MMAT was applied by the third author (CWB). The first author (CCL) checked the first 15% of included studies and, based on reaching 100% agreement on the application of the rating criteria, the primary reviewer then applied the tool independently to the remaining studies.

**Table 4:** MMAT

	Bardosh et al. 2017 [16]	Brewster et al. 2015 [17]	Carrera et al. 2015 [18]	Frykman et al. 2014 [19]	Wiener-Ogilvie et al. 2008 [20]	Atkins et al. 2008 [21]	Baer et al. 2009 [22]	Bonetti et al. 2005 [23]	Garner et al. 2011 [24]	Glisson et al. 2010 [25]	Holth et al. 2011 [26]	Lee et al. 2018 [27]	Lochman et al. 2009 [28]	Rapkin et al. 2017 [29]	Rohrbach et al. 1993 [30]	Seys et al. 2018 [31]	Williams et al. 2014 [32]	Williams et al. 2017 [33]		
1. Qualitative																				
Data sources relevant?	Y	Y	Y	Y	Y	N/A	N/A	N/A	N/A	N/A	N/A	N/A	N/A	N/A	N/A	N/A	N/A	N/A		
Data analysis process relevant?	Y	Y	Y	Y	Y	N/A	N/A	N/A	N/A	N/A	N/A	N/A	N/A	N/A	N/A	N/A	N/A	N/A		
Findings relate to context?	Y	Y	Y	Y	Y	N/A	N/A	N/A	N/A	N/A	N/A	N/A	N/A	N/A	N/A	N/A	N/A	N/A		
Findings relate to researchers' influence?	N	N	N	Y	N	N/A	N/A	N/A	N/A	N/A	N/A	N/A	N/A	N/A	N/A	N/A	N/A	N/A		
2. Quantitative randomized																				
Clear description of the randomization?	N/A	N/A	N/A	N/A	N/A	N	N	Y	Y	N	N	Y	N	Y	N	Y	N	Y		
Clear description of allocation or concealment?	N/A	N/A	N/A	N/A	N/A	N	N	N	N	Y	N	N	Y	N	Y	N	N	N		
Complete outcome data?	N/A	N/A	N/A	N/A	N/A	Y	Y	Y	Y	Y	Y	Y	Y	Y	N	Y	N	Y		
Low withdrawal/drop-out?	N/A	N/A	N/A	N/A	N/A	Y	Y	N	Y	N	N	N	Y	N	Y	N	Y	Y		
Total score (%)	75	75	75	100	75	50	50	50	75	50	25	50	75	50	50	50	25	25		
	Aarons et al. 2009 [34]	Becker et al. 2016 [35]	Beenstock et al. 2012 [36]	Beets et al. 2008 [37]	Bonetti et al. 2009 [38]	Chou et al. 2011 [39]	Cummings et al. 2017 [40]	David and Schiff 2017 [41]	Edmunds et al. 2014 [42]	Gnich et al. 2018 [43]	Guerrero et al. 2018 [44]	Huis et al. 2013 [45]	Little et al. 2015 [46]	Llasus et al. 2014 [47]	Nelson and Steele 2007 [48]	Potthoff et al. 2017 [49]	Presseau et al. 2016 [50]	Simmonds et al. 2012 [51]	Stockdale et al. 2018 [52]	Wanless et al. 2015 [53]
3. Quantitative - non-randomized																				
Recruitment minimizes selection bias?	Y	N	N	Y	Y	Y	N	N	Y	Y	Y	Y	Y	N	Y	Y	Y	Y	Y	Y
Measurements appropriate?	Y	Y	Y	Y	Y	Y	Y	Y	Y	Y	Y	Y	Y	Y	Y	Y	Y	Y	Y	Y
Comparable groups or control for differences?	Y	Y	Y	N	N	Y	N	Y	Y	Y	Y	N	Y	Y	Y	N	Y	Y	Y	Y
Complete outcome data, acceptable response rate, or acceptable follow-up rate?	N	N	N	N	N	N	N	Y	N	N	N	Y	Y	N	N	N	N	Y	N	N
Total score (%)	75	50	50	50	50	75	25	75	75	75	75	75	100	50	75	50	75	100	75	75
	Armson et al. 2018 [54]				Birken et al. 2015 [55]				Kauth et al. 2010 [56]				Lukas et al. 2009 [57]		Panzano et al. 2012 [58]		Rangachari et al. 2015 [59]		Shrubsole et al. 2018 [60]	
1. Qualitative																				
Data sources relevant?	Y				Y				Y				Y		Y		Y		Y	
Data analysis process relevant?	Y				Y				Y				N		N		Y		Y	
Findings relate to context?	Y				Y				Y				Y		Y		Y		Y	
Findings relate to researchers' influence?	N				N				N				N		N		N		N	
2. Quantitative randomized																				
Clear description of the randomization?	N/A				N/A				N				N/A		N/A		N/A		Y	
Clear description of allocation or concealment?	N/A				N/A				N				N/A		N/A		N/A		Y	
Complete outcome data?	N/A				N/A				Y				N/A		N/A		N/A		Y	
Low withdrawal/drop-out?	N/A				N/A				Y				N/A		N/A		N/A		N	
3. Quantitative non-randomized																				
Recruitment minimizes selection bias?	Y				Y				N/A				Y		N		Y		N/A	
Measurements appropriate?	Y				Y				N/A				Y		Y		Y		N/A	
Comparable groups or control for differences?	N				N				N/A				N		N		N		N/A	
Complete outcome data, acceptable response rate, or acceptable follow-up rate?	Y				N				N/A				N		Y		Y		N/A	
4. Mixed methods																				
Research design relevant?	Y				Y				Y				N		N		Y		Y	
Integration of qualitative and quantitative data relevant?	Y				Y				Y				Y		Y		Y		Y	
Appropriate consideration given to limitations associated with integration?	Y				Y				N				N		N		N		N	
Total score (%)	75				50				50				25		25		75		75	

### Data extraction and synthesis

Data extraction focused on several categories: study information/ background (i.e., country, setting, and sample), methods (i.e., theories that informed study, measures used, study design, analyses used, proposed mediation model), results (i.e., statistical relations between proposed variables of the mediation model tested), and criteria for establishing mechanisms (based on the seven listed in Table 2 [4];). All authors contributed to the development of data extraction categories that were applied to the full text of included studies. One reviewer (MRB) independently extracted relevant data and the other reviewer (CWB) checked the results for accuracy, with the first author (CCL) addressing any discrepancies or questions, consistent with the approach of other systematic reviews [61]. Extracted text demonstrating evidence of study meeting (or not meeting) each criterion for establishing a mechanism was further independently coded as “1” reflecting “criterion met” or “0” reflecting “criterion not met” by MRB and checked by CWB. Again, discrepancies and questions were resolved by the first author (CCL). Technically, mechanisms were considered “established” if all criteria were met. See Additional File 2 for PRISMA checklist for this study.

## Results

**Fig. 1:**
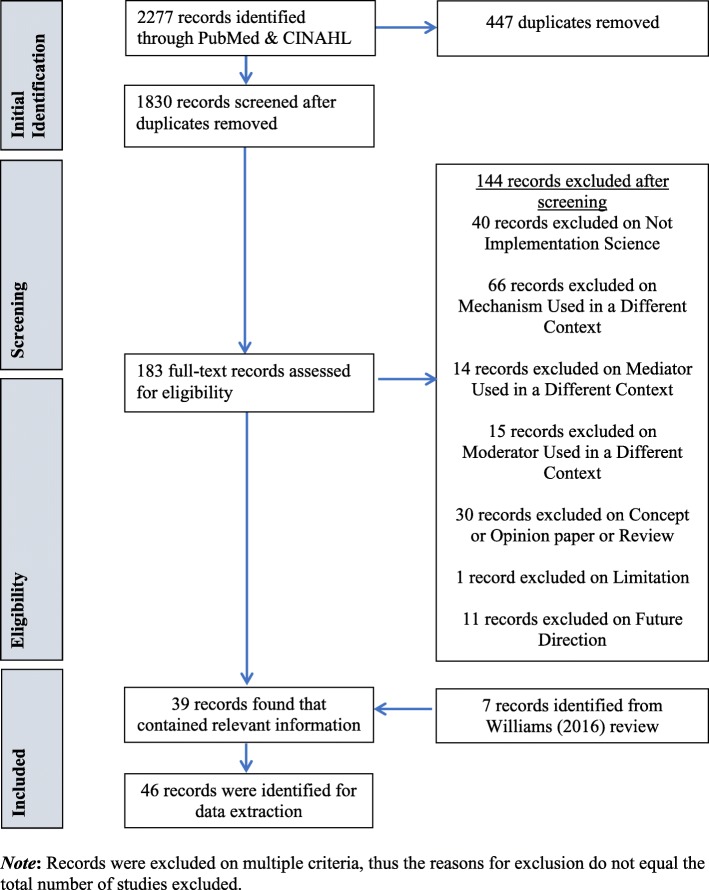
Mechanisms of Implementation Systematic Review PRISMA Flowchart

The search of PubMed and CINAHL Plus yielded 2277 studies for title and abstract screening, of which 447 were duplicates, and 183 moved on to full-text review for eligibility. Excluded studies were most frequently eliminated due to the use of mechanism in a different context (i.e., to refer to a process, technique, or system for achieving results of something other than implementation strategies). After full article review, 39 studies were deemed suitable for inclusion in this review. Two of the included studies appeared in the only other systematic review of implementation mechanisms in mental health settings [14]. For consistency and comprehensiveness, the remaining seven studies from the previously published review were added to the current systematic review for a total of 46 studies.^2^ See Fig. 1 for a PRISMA Flowchart of the screening process and results.

### Study characteristics

#### Setting, sampling, and interventions

Table 5 illustrates the characteristics of the 46 included studies. Twenty-five studies (54.3%) were completed in the USA, while 21 studies were conducted in other countries (e.g., Australia, Canada, Netherlands, UK). Settings were widely variable; studies occurred in behavioral health (e.g., community mental health, residential facilities) or substance abuse facilities most frequently (21.7%), followed by hospitals (15.2%), multiple sites across a health care system (15.2%), schools (15.2%), primary care clinics (10.9%), and Veteran’s Affairs facilities (8.7%). Sampling occurred at multiple ecological levels, including patients (17.4%), providers (65.2%), and organizations (43.5%). Seventeen (40.0%) studies examined the implementation of a complex psychosocial intervention (e.g., Cognitive behavioral therapy [42, 56];, multisystemic therapy [25, 26, 58]).

**Table 5:** Descriptive summary

Study	Setting	Sample	Intervention/Innovation	Complex psychosocial intervention	Design
Qualitative					
Bardosh et al. 2017 [16]	Health care facilities, multiple countries	Key informants (researchers, Mhealth staff, clinic staff, government officials; *n* = 32)	Mobile health application	N	Qualitative, cross sectional, comparative case study, non-randomized
Brewster et al. 2015 [17]	Hospitals	Hospitals (*k =* 10); hospital employees (hospital staff, *n =* 82; state hospital representatives *n =* 8)	Initiative to reduce rehospitalization rates	N	Qualitative, descriptive, cross sectional, non-randomized
Carrera and Lambooij 2015 [18]	Primary care	Patients (*n =* 12); health care providers (*n =* 4)	Blood pressure monitoring guidelines	N	Qualitative descriptive, cross sectional, non-randomized
Frykman et al. 2014 [19]	Emergency departments	Departments (*k =* 2), health care providers (*n =* 11)	Multi-professional teamwork guideline	N	qualitative, longitudinal (2 assessment points, 21 months), comparative case study, non-randomized
Wiener-Ogilvie et al. 2008 [20]	Primary care	Health care providers (*n =* 9)	Asthma management guideline	N	qualitative, cross sectional, comparative case study, non-randomized
Quantitative randomized					
Atkins et al. 2008 [21]	Schools	Teachers (*n =* 127); mental health providers (*n =* 21)	Attention Deficit Hyperactivity Disorder guidelines	Y	quantitative, longitudinal (5 assessment points, 2 years), randomized
Baer et al. 2009 [	Substance abuse treatment facilities	Substance abuse treatment facilities (*k =* 6); Mental health providers (*n =* 118)	Motivational Interviewing	Y	quantitative, longitudinal (3 assessment points, 6 months), randomized
Bonetti et al. 2005 [23]	Primary care	Health care providers (*n =* 152)	Spinal X-ray referral guidelines	N	quantitative, longitudinal (2 assessment points, 2 months), randomized control trial
Garner et al. 2011 [24]	Substance abuse treatment facilities	Substance abuse treatment facilities (*k =* 29); mental health providers (*n =* 95)	Adolescent Community Reinforcement Approach and Assertive Continuing Care	Y	quantitative, longitudinal (2 assessment points, 3 years), randomized control trial
Glisson et al. 2010 [25]	Juvenile courts	Counties (*k =* 14); patients (*n =* 615)	Multisystemic Therapy	Y	quantitative, longitudinal (weekly, quarterly, 4 years), randomized control trial
Holth et al. 2011 [26]	Behavioral health facilities	Mental health providers (*n =* 21); families (youth and primary caregiver; *n =* 41)	Multisystemic Therapy, Cognitive Behavior Therapy	Y	quantitative, longitudinal (monthly, 17 months), block randomized control trial
Lee et al. 2018 [27]	Schools, child care facilities	Organizations (*n =* 121)	Nutritional guidelines	N	quantitative, longitudinal (two time points; 2 studies at 6 months, 1 study at 12 months), analysis of aggregated datasets from three randomized control trials
Lochman et al. 2009 [28]	Schools	Schools (*k =* 57); patients (*n =* 531); mental health providers (*n =* 49)	Coping Power Program	Y	quantitative, longitudinal (2 assessment points, 2 years), randomized
Rapkin et al. 2017 [29]	Public library system	Communities (*k =* 20); community members (*n =* 9374)	Cancer screening and prevention education programs	N	quantitative, randomized, stepped-wedge, longitudinal
Rohrbach et al. 1993 [30]	Schools	Schools (*k =* 25); administrators (*n =* 25); teachers (*n =* 60); patients (*n =* 1147)	Adolescent Alcohol Prevention Trial	Y	quantitative, longitudinal (3 assessment points, 2 years), randomized control trial
Seys et al. 2018 [31]	Hospitals	Care teams (*k =* 19); care team members (*n =* 284); patients (*n =* 257)	Care pathway for Chronic Obstructive Pulmonary Disease	N	quantitative, longitudinal (two assessment points, 30 days), randomized
Williams et al. 2014 [32]	Behavioral health facilities	Behavioral health facilities (*k =* 92); administrators (*n =* 311)	Motivational Interviewing	Y	quantitative, longitudinal (3 assessment points, 3 months), randomized control trial
Williams et al. 2017 [33]	Behavioral health facilities	Organizations (*k =* 14); clinicians (*n =* 475)	Evidence-based practice (not specified)	Evidence-based practice implemented not reported	quantitative, longitudinal, randomized (4 assessment points, 4 years)
Quantitative non-randomized					
Aarons et al. 2009 [34]	Behavioral health facilities	Mental health care providers (*n =* 174)	31 child or family evidence-based practices	Y^a^	quantitative, cross-sectional, survey, non-randomized
Becker et al. 2016 [35]	Substance abuse treatment facilities	Clinics (*k =* 15); treatment providers (*n =* 60)	Contingency management treatment	Y	quantitative, longitudinal (biweekly, 12 months), non-randomized
Beenstock et al. 2012 [36]	Hospitals	Hospitals (*k =* 8); health care providers (*n =* 364)	Smoking cessation guideline	N	quantitative, cross sectional, survey, non-randomized
Beets et al. 2008 [37]	Schools	Teachers (*n* time 1 *=* 171, *n* time 2 *=* 191)	Positive Action Program	Y	quantitative, cross sectional at two time points, survey, non-randomized
Bonetti et al. 2009 [38]	Dentist offices	Health care providers (*n =* 133)	Fissure sealant evidence-based practice	N	quantitative, longitudinal, predictive cohort study (3 assessment points, 28 months), non-randomized
Chou et al. 2011 [39]	Veterans Affairs	Hospitals (*k =* 132), health care providers (*n =* 2,438)	Major depressive disorder screening guideline	N	quantitative, cross sectional, survey, randomized
Cummings et al. 2017 [40]	Nursing homes	Nursing homes (*k =* 7); nursing home staff (*n =* 333)	Coaching for Impressive Care	N	quantitative, , non-randomized two-group crossover
David and Schiff 2017 [41]	Health care system, multiple sites	Health care providers (*n =* 77)	Child-Parent Psychotherapy	Y	quantitative, cross sectional, survey, non-randomized
Edmunds et al. 2014 [42]	Behavioral health facilities	Mental health providers (*n =* 50)	Cognitive Behavioral Therapy	Y	quantitative, longitudinal, non-randomized
Gnich et al. 2018 [43]	Dentist offices	Health care providers (*n =* 709)	Fluoride varnish application	N	quantitative, longitudinal (2 assessment points, 18 months), non-randomized
Guerrero et al. 2018 [44]	Behavioral health facilities	Behavioral health facilities (*k =* 112), mental heal providers (*n =* 427)	Contingency management treatment and medicationassisted treatment	Y	quantitative, longitudinal (2 assessment points), survey, non-randomized
Huis et al. 2013 [45]	Hospitals	Hospitals (*k =* 3); departments (*k =* 67): health care providers (*k =* 2733)	Hand hygiene guidelines	N	quantitative, longitudinal, process evaluation of a cluster randomized controlled trial
Little et al. 2015 [46]	Schools	School districts (*k =* 183); departments (*k =* 22)	Tobacco Use Prevention Education	N	quantitative, cross sectional, survey, non-randomized
Llasus et al. 2014 [47]	University nursing programs	Nursing students (*n =* 174)	Evidence-based practices (not specified)	N	quantitative, descriptive, correlational, cross sectional, survey, non-randomized
Nelson and Steele 2007 [48]	Health care system, multiple sites	Mental health providers (*n =* 214)	Evidence-based practices (not specified)	N	quantitative, cross sectional, survey, non-randomized
Potthoff et al. 2017 [49]	Primary care	Organizations (*k =* 99); health care providers (*n =* 489)	Type 2 diabetes management guideline	N	quantitative, longitudinal (2 assessment points, 1 year), correlational, survey, non-randomized
Presseau et al. 2016 [50]	Primary care	Family physicians (time 1 *n =* 632; time 2 *n =* 426)	Prescription of hypertension medication	N	quantitative, longitudinal (2 assessment points, approximately 8 months), 2X3 factorial
Simmonds et al. 2012 [51]	Health care system, multiple sites	Health care providers (*n =* 108)	Lower back pain management guidelines	N	quantitative, cross sectional, survey, non-randomized
Stockdale et al. 2018 [52]	Veterans Affairs	Health care providers (*n =* 149), patients (*n =* 3329)	Patient Centered Medical Home	N	quantitative, cross sectional, survey, non-randomized
Wanless et al. 2015 [53]	Schools	Schools (*k =* 13); teachers (*n =* 1114)	Responsive Classroom	Y	quantitative, longitudinal, non-randomized (focuses on one condition in an RCT)
Yamada et al. 2017 [62]	Hospitals	Care units (*k =* 32); nurses (*n =* 779); patients (*n =* 1,604)	Instrumental and conceptual research use, evidence-based pain assessment	N	quantitative, cross sectional, non-randomized
Mixed Methods					
Armson et al. 2018 [54]	Health care system, multiple sites	Health care providers (*n =* 70)	Breast cancer screening guideline	N	mixed method, longitudinal, observational/ naturalist field study, non-randomized
Birken et al. 2015 [55]	Health care system, multiple sites	Organizations (*k =* 149); administrators (*n =* 223)	Quality improvement initiative based on Chronic Care Model	N	mixed method sequential, cross sectional, non-randomized
Kauth et al. 2010 [56]	Veterans Affairs	Clinics (*k =* 21); mental health providers (*n =* 23)	Cognitive Behavioral Therapy	Y	mixed method, quasi-experimental, longitudinal (2 assessment points, 6 months), randomized
Lukas et al. 2009 [57]	Veterans Affairs	Organizations (*k =* 78); health care providers, non-clinical staff (*n =* 3870)	Advance Clinic Access	N	mixed method, cross sectional, observational, non-randomized
Panzano et al. 2012 [58]	Behavioral health facilities	Consultants (*n =* 34); mental health providers (*n =* 70)	Multisystemic Therapy, Dual Disorder Treatment, Ohio medication algorithms, Cluster-based Outcomes Management	Y	mixed method, longitudinal, observational/ naturalist field study, non-randomized
Rangachari et al. 2015 [59]	Hospitals	Departments (k *=* 2); health care providers (*n =* 101); administrators (*n =* 6)	Central line bundle catheter insertion evidence-based practice	N	prospective, longitudinal, exploratory field study, mixed-method analysis
Shrubsole et al. 2018 [60]	Hospitals	Hospitals (*k =* 4); health care providers (*n =* 37); patients (*n =* 107)	Aphasia management practices	N	mixed method, longitudinal, cross-over, cluster randomized control trial

^a^Multiple EBPs, some of which were complex psychosocial interventions

#### Study design

Our review included six qualitative (10.9%), seven mixed methods (15.2%), and 34 quantitative studies (73.9%). The most common study design was quantitative non-randomized/observational (21 studies; 45.7%), of which 11 were cross-sectional. There were 13 (28.3%) randomized studies included in this review. Twenty-nine studies (63.0%) were longitudinal (i.e., included more than one data collection time point for the sample).

### Study quality

Table 4 shows the results of the MMAT quality assessment. Scores for the included studies ranged from 25 to 100%. Six studies (13.0%) received a 25% rating based on the MMAT criteria [15], 17 studies (40.0%) received 50%, 21 studies (45.7%) received 75%, and only three studies (6.5%) scored 100%. The most frequent weaknesses were the lack of discussion on researcher influence in qualitative and mixed methods studies, lack of clear description of randomization approach utilized in the randomized quantitative studies, and subthreshold rates for acceptable response or follow-up in non-randomized quantitative studies.

#### Study design and evaluation of mechanisms theories, models, and frameworks

Twenty-seven (58.7%) of the studies articulated their plan to evaluate mechanisms, mediators, or moderators in their research aims or hypotheses; the remaining studies included this as a secondary analysis. Thirty-five studies (76.1%) cited a theory, framework, or model as the basis or rationale for their evaluation. The diffusion of innovations theory [63, 64] was most frequently cited, appearing in nine studies (19.6%), followed by the theory of planned behavior [65], appearing in seven studies (15.2%). The most commonly cited frameworks were the theoretical domains framework (five studies; 10.9%) [66] and Promoting Action on Research in Health Services (PARiHS) [67] (three studies; 6.5%).

#### Ecological levels

Four studies (8.7%) incorporated theories or frameworks that focused exclusively on a single ecological level; two focusing on leadership, one at the organizational level, and one at the systems level. There was some discordance between the theories that purportedly informed studies and the potential mechanisms of interest, as 67.4% of candidate mechanisms or mediators were at the intrapersonal level, while 30.4% were at the interpersonal level, and 21.7% at the organizational level. There were no proposed mechanisms at the systems or policy level. Although 12 studies (26.1%) examined mechanisms or mediators across multiple ecological levels, few explicitly examined multilevel relationships (e.g., multiple single-level mediation models were tested in one study).

#### Measurement and analysis

The vast majority of studies (38, 82.6%) utilized self-report measures as the primary means of assessing the mechanism, and 13 of these studies (28.3%) utilized focus groups and/or interviews as a primary measure, often in combination with other self-report measures such as surveys. Multiple regression constituted the most common analytic approach for assessing mediators or moderators, utilized by 25 studies (54.3%), albeit this was applied in a variety of ways. Twelve studies (26.1%) utilized hierarchical linear modeling (HLM) and six studies (13.0%) utilized structural equation modeling (SEM); see Table 6 for a complete breakdown. Studies that explicitly tested mediators employed diverse approaches including Baron and Kenny’s (*N* = 8, 17.4 causal steps approach [78], Preacher and Hayes’ (*N =* 3, 6.5%) approach to conducting bias-corrected bootstrapping to estimate the significance of a mediated effect (i.e., computing significance for the product of coefficients) [95, 126], and Sobel’s (*N =* 4, 8.9%) approach to estimating standard error for the product of coefficients often using structural equation modeling [79]. Only one study tested a potential moderator, citing Raudenbush’s [80, 82]. Two other studies included a potential moderator in their conceptual frameworks, but did not explicitly test moderation.

**Table 6:** Mechanism analysis

Study	Aims	Theory, framework, model	Mechanism measurement	Mediation testing citation
Qualitative				
Bardosh et al. 2017 [16]	N	Consolidated framework for implementation research [68]	Interviews	None
Brewster et al. 2015 [17]	Y	Implementation innovation framework [69]	Interviews	None
Carrera and Lambooij 2015 [18]	N	Technology acceptance model [70]; Theory of planned behavior [65]; Model of personal computing utilization [71]	Focus groups	None
Frykman et al. 2014 [19]	N	Direction, competence, opportunity and motivation (DCOM) [72, 73]	Interviews; observations	None
Wiener-Ogilvie et al. 2008 [20]	N	None reported	Interviews; focus groups	None
Quantitative- randomized				
Atkins et al. 2008 [21]	Y	Diffusion of innovation theory [63]	Interviews; self-report	[74]
Baer et al. 2009 [22]	Y	None reported	interviews; self-report	[75]
Bonetti et al. 2005 [23]	N	Theory of planned behavior [65]; Social cognitive theory [76, 77]	Self-report	[78, 79]
Garner et al. 2011 [24]	N	Theory of planned behavior [65]	Self-report	[80, 81]
Glisson et al. 2010 [25]	N	None reported	Self-report, audiotape coding and interviews	[82]
Holth et al. 2011 [26]	Y	None reported	Interviews; self-report	[83]
Lee et al. 2018 [27]	Y	Theoretical domains framework [84]	Self-report, secondary analysis	[85, 86]
Lochman et al. 2009 [28]	N	Diffusion of innovation theory [87]	Coder ratings	[88]
Rapkin et al. 2017 [29]	Y	None reported	Self-report	[89]
Rohrbach et al. 1993 [30]	N	Diffusion of innovation theory [64]	Interviews; self-report; observations	None
Seys et al. 2018 [31]	Y	None reported	Chart review; self-report	[78]
Williams et al. 2014 [32]	Y	Diffusion of innovation theory [87]	Self-report	[90, 91]
Williams et al., 2017 (66)	Y	Organizational culture theory [32] and Theory of planned behavior [65]	Self-report	[92]
Quantitative- non-randomized				
Aarons et al. 2009 [34]	Y	Institutional theory [93], Theory of planned behavior [65], Theory of perceived organizational support [94]	Self-report	[78]
Becker et al. 2016 [35]	Y	Diffusion of innovation theory [64]	Self-report	None
Beenstock et al. 2012 [36]	N	Theoretical domains framework [66]	Self-report	[95]
Beets et al. 2008 [37]	Y	Theory driven evaluation [96]; Diffusion of innovation theory [63]	Self-report	[97, 98]
Bonetti et al. 2009 [38]	N	Theory of planned behavior [65]; Social cognitive theory [99]; Operant learning theory [100]; Action planning [101]; Common sense self-regulation model [102]; Precaution adoption process model [103]; Stage theory [103, 104]	Self-report; objective measure	[78, 79]
Chou et al. 2011 [39]	N	Goal setting theory [105]; Goal commitment theory [106]	Self-report	[80, 107]
Cummings et al. 2017 [40]	N	Promoting action on research in health services (PARiHS) [67]	Self-report	[108]
David and Schiff 2017 [41]	Y	Diffusion of innovation theory [87, 109]	Self-report	[110]
Edmunds et al. 2014 [42]	Y	EPIS framework [111]	Self-report	[80, 112]
Gnich et al. 2018 [43]	Y	Theoretical domains framework [66]	Self-report	None
Guerrero et al. 2018 [44]	Y	Theory on middle manager s[69]	Self-report	[113]
Huis et al. 2013 [45]	N	None reported	Observations; self-report; website visitor registration; logs; field Notes; effect evaluation; quiz	none
Little et al. 2015 [46]	N	Diffusion of innovation theory [64]	Self-report	[114–116]
Llasus et al. 2014 [47]	N	Knowledge to action conceptual framework [117]	Self-report	[78, 79, 95]
Nelson and Steele 2007 [48]	N	None reported	Self-report	None
Potthoff et al. 2017 [49]	Y	Dual process model of behavior [118]	Self-report	[79]
Presseau et al. 2016 [50]	Y	Theory of planned behavior [65]	Self-report	None
Simmonds et al. 2012 [51]	Y	None reported	Self-report	[78]
Stockdale et al. 2018 [52]	Y	None reported	Self-report	[119]
Wanless et al. 2015 [53]	Y	None reported	Self-report, observation	[110]
Yamada et al. 2017 [62]	Y	Promoting action on research in health services (PARiHS) [120]	Self-report, chart review	None
Mixed methods				
Armson et al. 2018 [54]	Y	Theoretical domains framework [66]	Interviews; self-report	None
Birken et al. 2015 [55]	N	Hierarchical taxonomy of leader behavior [121]	Interviews; self-report	[95, 122]
Kauth et al. 2010 [56]	Y	Fixsen model [123]; Promoting action on research in health services (PARiHS) [120]	Self-report; logs	None
Lukas et al. 2009 [57]	Y	Diffusion of Innovations Theory [63, 124]	Interviews	[78]
Panzano et al. 2012 [58]	Y	None reported	Self-report	[78]
Rangachari et al. 2015 [59]	N	Complex systems theory [125]	Infection rate; chart review; hospital records; logs	None
Shrubsole et al. 2018 [60]	N	Theoretical domains framework [66]	Chart review; self-report	none

### Emergent mechanism models

There was substantial variation in the models that emerged from the studies included in this review. Table 7 represents variables considered in mediating or moderating models across studies (or identified as candidate mediators, moderators, or mechanisms in the case of qualitative studies). Additional file 3 depicts the unique versions of models tested and their associated studies. We attempted to categorize variables as either (a) an independent variable (*X*) impacting a dependent variable; (b) a dependent variable (*Y*), typically the outcome of interest for a study; or (c) an intervening variable (*M*), a putative mediator in most cases, though three studies tested potential moderators. We further specified variables as representing a strategy, determinant, and outcome; see Table 1 for definitions.^3^

**Table 7:** *Model tested*

Study	Independent variable (*X*)	Intervening variable (*M*)	Dependent variable (*Y*)
Qualitative			
Bardosh et al. 2017 [16]	Mobile and text follow up with patients	Service organization at clinic level, clinician norms and practices, availability of local champions staff, adaptability and co-design of strategy, receptivity and capacity of local management	Culture of care
Brewster et al. 2015 [17]	Patient education, follow-up phone calls to patients after discharge, discharge planning, collaboration with post-acute providers	Intrinsic reward to staff --> shift in norms and attitudes	Reduced hospital readmissions
Carrera and Lambooij 2015 [18]	None reported	Mediators: perceived usefulness, perceived ease of use, self-efficacy, attitudes,social norm Moderator: enabling conditions	Intervention acceptability (providers and patients)
Frykman et al. 2014 [19]	Senior manager and consultant-driven teamwork strategy, senior manager and staff-driven teamwork strategy	Direction, communication, opportunity, motivation	Change in staff behavior
Wiener-Ogilvie et al. 2008 [20]	Guideline implementation	Practice organization (delegation of work to nurses)	Compliance with guidelines
Quantitative—randomized			
Atkins et al. 2008 [21]	Training and consultation	Key opinion leader instrumental supportmental health professional instrumental support	Teacher self-reported used of ADHD guidelines
Baer et al. 2009 [22]	Climate for organizational change	Post training agency activities to support use of Motivational Interviewing	Fidelity to intervention (Motivational Interviewing spirit and response to question ratio)
Bonetti et al. 2005 [23]	Audit and feedback	Decision difficulty, behavioral control	Simulated behavior
Garner et al. 2011 [24]	Pay for performance	1. Subjective norms 2. Attitudes toward intervention 3. Perceived control	1. Therapists’ intention to achieve monthly competence 2. Therapists’ intention to achieve targeted threshold
Glisson et al. 2010 [25]	Availability responsiveness and continuity (ARC) Intervention + Multisystemic Therapy quality assurance, pay for performance	Fidelity to multisystemic therapy	Rate of change in child behavior out of home placements
Holth et al. 2011 [26]	Workshop + manual, intensive quality assurance + workshop + manual	Adherence to contingency management and cognitive behavioral therapy techniques	Youth cannabis use
Lee et al. 2018 [27]	Implementation strategy bundles (varied across studies)	Knowledge, skills, social/professional role and identity, environmental resources	Nutrition guideline implementation
Lochman et al. 2009 [28]	Intensive training + feedback, basic training	# of sessions attended, # of objectives completed, # of contacts with trainers, counselor engagement w/clients	Client externalizing behaviors, client social skills, client study skills, client expectancies re: aggression, consistent parenting, client assaultive acts
Rapkin et al. 2017 [29]	Indicators of program activities: cumulative local programs, attendance at local programs, time since most recent local program, personal awareness of programs, cumulative outside programs	Mediators: awareness of free/low cost cancer screening, cancer knowledge, cancer information seeking, having health insurance, annual physical moderator: frequency of library use	Cancer screening attempts to quit smokingtobacco cessation
Rohrbach et al. 1993 [30]	1. Teacher training 2. Principal support intervention	1a. Teacher self-efficacy, 1b. enthusiasm, 1c. preparedness 2a. Principal encouragement, 2b. Principal beliefs about program	Quantity of program implementation
Seys et al. 2018 [31]	Care pathway implementation	Adherence to evidence-based recommendations, level of competence, team climate for innovation, burnout, level of organized care	30-day hospital readmission
Williams et al. 2014 [32]	Information packets and Motivational Interviewing webinar	Attitudes towards EBPs, pressure for change, barriers to EBPs, resources, organizational climate, management support	Motivational Interviewing adoption
Williams et al. 2017 [33]	Availability,Responsiveness, and Continuity (ARC) intervention implementation	Proficiency culture --> evidence-based practice intention, barrier reduction	EBP adoption, EBP use
Quantitative—non-randomized			
Aarons et al. 2009 [34]	Agency type	1. organizational support for EBP --> provider attitudes towards EBP 2, 3 organizational support for EBP organizational support for EBP	1,3 provider EBP use2. provider EBP attitudes
Becker et al. 2016 [35]	Training as usual, training + ongoing technical assistance, support from in-house champion, specialized training on change process, monthly conference calls and online forum to support change	Organizational readiness to change (motivation for change, adequacy of resources, staff attributes, organizational climate),perceived intervention characteristics (relative advantage, observability, trialability, compatibility, and complexity)	Adoption
Beenstock et al. 2012 [36]	Main place of work	Propensity to act	Referral of women to smoking cessation services
Beets et al. 2008 [37]	Perception of school climate	1. Beliefs about responsibility to teach program 2. beliefs about responsibility to teach program --> attitudes towards program --> curriculum delivered	1. Attitudes towards program 2. curriculum delivered to schoolwide material usage
Bonetti et al. 2009 [38]	Behavioral intention	Action planning	Placing fissure sealants
Chou et al. 2011 [39]	Receipt of individual performance feedback, clinician input into guideline implementation and quality improvement, clinician expectancy, clinician self-efficacy	Agreement with guidelines, adherence to guidelines, improved knowledge, practice delivery	Fidelity to screening patients for depression
Cummings et al. 2017 [40]	Culture, feedback, leadership and resources	Manager support, coaching conversations, job satisfaction	Conceptual research use, persuasive research use, instrumental research use
David and Schiff 2017 [41]	Child-parent psychotherapy social network Child-parent psychotherapy supervision	Self-efficacy	Number of child-parent psychotherapy cases, intention to use child-parent psychotherapy
Edmunds et al. 2014 [42]	Time following training	Time spent in consultation	Knowledge of cognitive behavioral therapy for anxiety, attitudes towards EBPs
Gnich et al. 2018 [43]	Pay-per item financial incentive	Knowledge, skills, social/professional role and identity, beliefs about consequences, motivation and goals (intention), environmental context and resources, social influences (norms), emotion, behavioral regulation	Fluoride varnish delivery
Guerrero et al. 2018 [44]	Top manager transformational leadership	Middle managers’ implementation leadership	Employee attitudes towards EBPs, EBP implementation
Huis et al. 2013 [45]	individual and organization targeted strategies (education, reminders, feedback), individual and organizational targeted strategies + team and leader strategy	Social influence, leadership, performance feedback	Handwashing fidelity
Little et al. 2015 [46]	Community priority, organizational support, program champion	beliefs about effectiveness of interventions --> funding to adopt program	Adoption
Llasus et al. 2014 [47]	EBP knowledge	Self confidence in one's EBP competencies (defined as readiness)	EBP implementation behaviors
Nelson and Steele 2007 [48]	EBP training, openness of clinical setting to EBPs	Positive attitudes towards treatment research, negative attitudes towards treatment research	EBP use
Potthoff et al. 2017 [49]	Action planning, coping planning	Habit	Clinical behaviors (prescribing, advising, examining)
Presseau et al. 2016 [50]	Printed informational materials	Attitudes toward prescribing, subjective norms, perceived behavioral control, intention to prescribe	Self-reported prescribing behavior
Simmonds et al. 2012 [51]	Intolerance of uncertainty	Treatment orientation toward back pain	Recommendations to return to work 2. recommendations to return to usual activities,estimated risk of back pain disability
Stockdale et al. 2018 [52]	Health care team communication	Patient-provider communication	Patient satisfaction with primary care provider
Wanless et al. 2015 [53]	Use of responsive classroom practices, global emotional support, self-efficacy, collective responsibility	Teacher training engagement	Fidelity to intervention
Yamada et al. 2017 [62]	Instrumental research use, conceptual research use	Organizational context: leadership, culture, evaluation, social capital, informal interactions, formal interactions, resources, slack space, slack staff, slack time	Pain assessment, evidence-based pain procedure use, pain intensity
Mixed methods			
Armson et al. 2018 [54]	Implementation tools (printed education materials, informational video, decision aid)	Evidence-based information in guideline, evidence-based information in screening module, discussions with peers, application of implementation tools, discussions with patients, lack of evidence about benefits, patients' screening expectations, fear of misdiagnosis, problems with having patient materials available	Use of breast cancer screening guidelines
Birken et al. 2015 [55]	1. Top manager support 2. Performance reviews 3. Human resources	Mediators: 1a. Performance reviews 1b. Human resources 1c. Training 1d. Funding 1e. Local social network involvement Moderator: 2/3. top manager support	1, 2, 3 middle manager commitment to innovation
Kauth et al. 2010 [56]	Facilitation + workshop, workshop	Job-related barriers, # of contacts with facilitator, time spent in facilitation	% time conducting Cognitive Behavioral Therapy
Lukas et al. 2009 [57]	Higher management support, group culture, hierarchical culture	Team effectiveness	Extent of implementation
Panzano et al. 2012 [58]	1. Strategic fit of intervention 2. Climate for innovation	1. Climate for innovation2. Fidelity to intervention	1. Fidelity to intervention 2. Assimilation
Rangachari et al. 2015 [59]	Emails containing intervention information and unit level adherence feedback + brief weekly training	Proactive communication between nurses and physicians emergence of champions	Number of catheter days
Shrubsole et al. 2018 [60]	Tailored training intervention targeting information provision	Mechanisms of Intervention 1 targeting information provision implementation): knowledge, beliefs about consequences, social influence, beliefs about capabilities, environmental context and resources Mechanisms of Intervention 2 targeting implementation of goal setting): beliefs about consequences, social influences, beliefs about capabilities, environmental context and resources	Information provisiongoal setting

Numbering is used to denote match variables across models; not all models tested the same sets of variables

#### Common model types

The most common model type (29; 63.0%) was one in which *X* was a determinant, *M* was also a determinant, and *Y* was an implementation outcome variable (determinant ➔ determinant ➔ implementation outcome). For example, Beenstock et al. [36] tested a model in which propensity to act (determinant) was evaluated as a mediator explaining the relation between main place of work (determinant) and referral to smoking cessation services (outcome). Just less than half the studies (22; 47.8%) included an implementation strategy in their model, of which 16 (34.8%) evaluated a mediation model in which an implementation strategy was *X*, a determinant was the candidate *M*, and an implementation outcome was *Y* (strategy ➔ determinant ➔ implementation outcome); ten of these studies experimentally manipulated the relation between the implementation strategy and determinant. An example of this more traditional mediation model is a study by Atkins and colleagues [21] which evaluated key opinion leader support and mental health practitioner support (determinants) as potential mediators of the relation between training and consultation (strategy) and adoption of the EBP (implementation outcome). Five studies included a mediation model in which *X* was an implementation strategy, *Y* was a clinical outcome, and *M* was an implementation outcome (strategy ➔ implementation outcome ➔ clinical outcome) [25, 26, 28, 29, 31].

#### Notable exceptions to model types

While the majority of quantitative studies tested a three-variable model, there were some notable exceptions. Several studies tested multiple three variable models that held the independent variable and mediator constant but tested the relation among several dependent variables. Several studies tested multiple three variable models that held the independent variable and dependent variables constant but tested several mediators.

#### Qualitative studies

Five studies included in this review utilized qualitative methods to explore potential mechanisms or mediators of change, though only one explicitly stated this goal in their aims [17]. Three studies utilized a comparative case study design incorporating a combination of interviews, focus groups, observation, and document review, whereas two studies employed a cross-sectional descriptive design. Although three of the five studies reported their analytic design was informed by a theory or previously established model, only one study included an interview guide in which items were explicitly linked to theory [19]. All qualitative studies explored relations between multiple ecological levels, drawing connections between intra and interpersonal behavioral constructs and organization or system level change.

### Criteria for establishing mechanisms of change

Finally, with respect to the seven criteria for establishing mechanisms of change, the plausibility/coherence (i.e., a logical explanation of how the mechanism operates that incorporates relevant research findings) was the most frequently fulfilled requirement, met by 42 studies (91.3%). Although 20 studies (43.5%), of which 18 were quantitative, provided statistical evidence of a strong association between the dependent and independent variables, only 13 (28.2%) studies experimentally manipulated an implementation strategy or the proposed mediator or mechanism. Further, there was only one study that attempted to demonstrate a dose-response relation between mediators and outcomes. Most included studies (39; 84.8%) fulfilled three or fewer criteria, and only one study fulfilled six of the seven requirements for demonstrating a mechanism of change; see Table 8.

**Table 8:** Kazdin criteria

	Association	Specificity	Consistency	Manipulation	Timeline	Gradient	Plausibility	Total
Qualitative								
Bardosh et al. 2017 [16]	0	0	1	0	0	0	1	2
Brewster et al. 2015 [17]	0	0	1	0	0	0	1	2
Carrera and Lambooij 2015 [18]	0	0	0	0	0	0	1	1
Frykman et al. 2014 [19]	0	0	0	0	1	0	1	2
Wiener-Ogilvie et al. 2008 [20]	0	0	1	0	0	0	1	2
Quantitative—randomized								
Atkins et al. 2008 [21]	0	0	1	1	0	0	1	3
Baer et al. 2009 [22]	0	0	1	0	1	0	1	3
Bonetti et al. 2005 [23]	1	1	1	0	1	0	1	5
Garner et al. 2011 [24]	0	1	0	1	0	0	1	3
Glisson et al. 2010 [25]	0	0	0	1	1	0	1	3
Holth et al. 2011 [26]	1	0	1	1	1	0	1	4
Lee et al. 2018 [27]	0	0	0	0	0	0	1	1
Lochman et al. 2009 [28]	0	1	0	1	1	0	0	3
Rapkin et al. 2017 [29]	1	0	0	0	1	1	1	4
Rohrbach et al. 1993 [30]	0	0	0	1	1	0	1	3
Seys et al. 2018 [31]	1	1	0	1	1	0	1	5
Williams et al. 2014 [32]	0	1	0	1	1	0	1	4
Williams et al. 2017 [33]	1	1	1	1	1	0	1	6
Quantitative—non-randomized								
Aarons et al. 2009 [34]	1	0	1	0	0	0	1	4
Becker et al. 2016 [35]	0	0	0	1	1	0	1	3
Beenstock et al. 2012 [36]	1	0	0	0	0	0	0	1
Beets et al. 2008 [37]	1	0	1	0	0	0	1	3
Bonetti et al. 2009 [38]	1	0	1	0	0	0	1	3
Chou et al. 2011 [39]	1	0	0	0	0	0	1	2
Cummings et al., 2017 [40]	0	0	1	0	0	0	1	3
David and Schiff 2017 [41]	1	0	1	0	0	0	1	3
Edmunds et al. 2014 [42]	0	0	0	0	1	0	0	1
Gnich et al. 2018 [43]	0	0	1	0	1	0	1	3
Guerrero et al. 2018 [44]	1	0	1	0	0	0	1	2
Huis et al. 2013 [45]	0	0	0	1	1	0	1	3
Little et al. 2015 [46]	1	0	0	0	0	0	1	2
Llasus et al. 2014 [47]	1	0	1	0	0	0	1	3
Nelson and Steele 2007 [48]	1	0	0	0	0	0	1	2
Potthoff et al. 2017 [49]	1	0	0	0	0	0	1	2
Presseau et al. 2016 [50]	0	0	1	0	0	0	1	2
Simmonds et al. 2012 [51]	1	0	0	0	0	0	1	2
Stockdale et al. 2018 [52]	1	0	0	0	0	0	1	2
Wanless et al. 2015 [53]	0	0	0	0	1	0	1	2
Mixed methods								
Armson et al. 2018 [54]	0	0	1	0	0	0	1	2
Birken et al. 2015 [55]	0	0	0	0	0	0	1	1
Kauth et al. 2010 [56]	0	0	0	1	1	0	1	3
Lukas et al. 2009 [57]	1	1	0	0	0	0	1	3
Panzano et al. 2012 [58]	1	0	0	0	0	0	1	2
Rangachari et al. 2015 [59]	0	0	0	0	1	0	0	1
Shrubsole et al. 2018 [60]	0	0	0	1	1	0	1	3

Studies that only tested mediation relationships are not included in this table

## Discussion

### Observations regarding mechanistic research in implementation science

Mechanism-focused implementation research is in an early phase of development, with only 46 studies identified in our systematic review across health disciplines broadly. Consistent with the field of implementation science, no single discipline is driving the conduct of mechanistic research, and a diverse array of methods (quantitative, qualitative, mixed methods) and designs (e.g., cross-sectional survey, longitudinal non-randomized, longitudinal randomized, etc.) have been used to examine mechanisms. Just over one-third of studies (*N =* 16; 34.8%) evaluated a mediation model with the implementation strategy as the independent variable, determinant as a putative mediator, and implementation outcome as the dependent variable. Although this was the most commonly reported model, we would expect a much higher proportion of studies testing mechanisms of implementation strategies given the ultimate goal of precise selection of strategies targeting key mechanisms of change. Studies sometimes evaluated models in which the determinant was the independent variable, another determinant was the putative mediator, and an implementation outcome was the dependent variable (*N =* 11; 23.9%). These models suggest an interest in understanding the cascading effect of changes in context on key outcomes, but without manipulating or evaluating an implementation strategy as the driver of observed change. Less common (only 5, 10.9%) were more complex models in which multiple mediators and outcomes and different levels of analyses were tested (e.g., [37, 39]), despite that this level of complexity is likely to characterize the reality of typical implementation contexts. Although there were several quantitative studies that did observe significant relations pointing toward a mediator, none met all criteria for establishing a mechanism.

Less than one-third of the studies experimentally manipulated the strategy-mechanism linkage. As the field progresses, we anticipate many more tests of this nature, which will allow us to discern how strategies exert their effect on outcomes of interest. However, implementation science will continue to be challenged by the costly nature of the type of experimental studies that would be needed to establish this type of evidence. Fortunately, methodological innovations that capitalize on recently funded implementation trials to engage in multilevel mediation modeling hold promise for the next iteration of mechanistic implementation research [14, 127] As this work unfolds, a number of scenarios are possible. For example, it is likely the case that multiple strategies can target the same mechanism; that a single strategy can target multiple mechanisms; and that mechanisms across multiple levels of analysis must be engaged for a given strategy to influence an outcome of interest. Accordingly, we expect great variability in model testing will continue and that more narrowly focused efforts will remain important contributions so long as shared conceptualization of mechanisms and related variables is embraced, articulated, and rigorously tested. As with other fields, we observed great variability in the degree to which mechanisms (and related variables of interest) were appropriately specified, operationalized, and measured. This misspecification coupled with the overall lack of high-quality studies (only three met 100% of the quality criteria), and the diversity in study methods, strategies tested, and mediating or moderating variables under consideration, we were unable to synthesize the findings across studies to point toward promising mechanisms.

### The need for greater conceptual clarity and methodological advancements

Despite the important advances that the studies included in this review represent, there are clear conceptual and methodological issues that need to be addressed to allow future research to more systematically establish mechanisms. Table 1 offers a list of key terms and definitions for the field to consider. We suggest the term “mechanism” be used to reflect a process or event through which an *implementation strategy* operates to affect desired *implementation outcomes*. Consistent with existing criteria [4], mechanisms can only be confidently established via carefully designed (i.e., longitudinal; experimentally manipulated) empirical studies demonstrating a strong association, and ideally a dose-response relation, between an intervening variable and outcome (e.g., via qualitative data or mediation or moderator analyses) that are supported by very specific theoretical propositions observed consistently across multiple studies. We found the term “mediator” to be most frequently used in this systematic review, which can point toward a mechanism, but without consideration of these full criteria, detection of a mediator reflects a missed opportunity to contribute more meaningfully to the mechanisms literature.

Interestingly, the nearly half of studies (43.5%) treated a variable that many would conceptualize as a “determinant” as the independent variable in at least one proposed or tested mediation pathway. Presumably, if researchers are exploring the impact of a determinant on another determinant and then on an outcome, there must be a strategy (or action) that caused the change in the initial determinant. Or, it is possible that researchers are simply interested in the natural associations among these determinants to identify promising points of leverage. This is a prime example where the variable or overlapping use of concepts (i.e., calling all factors of interest “determinants”) becomes particularly problematic and undermines the capacity of the field to accumulate knowledge across studies in the service of establishing mechanisms. We contend that it is important to differentiate among concepts to use more meaningful terms like preconditions, putative mechanisms, proximal and distal outcomes, all of which were under-specified in the majority of the included studies. Several authors from our team have articulated an approach to building causal pathway diagrams [128] that clarifies that preconditions are necessary factors for a mechanism to be activated and proximal outcomes are the immediate result of a strategy that is realized only because the specific mechanism was activated. We conceptualize distal outcomes as the eight implementation outcomes articulated by Proctor and colleagues [129]. Disentangling these concepts can help characterize why strategies fail to exert an impact on an outcome of interest. Examples of each follow in the section below.

### Conceptual and methodological recommendations for future research

#### Hypothesis generation

With greater precision among these concepts, the field can also generate and test more specific hypotheses about how and why key variables are related. This begins with laying out mechanistic research questions (e.g., How does a network intervention, like a learning collaborative, influence provider attitudes?) and generating theory-driven hypotheses. For instance, a testable hypothesis may be that learning collaboratives [strategy] operate through sharing [mechanism] of positive experiences with a new practice to influence provider attitudes [outcome]. As another example, clinical decision support [strategy] may act through helping the provider to remember [mechanism] to administer a screener [proximal outcome] and flagging this practice before an encounter may not allow the mechanism to be activated [precondition]. Finally, organizational strategy development [strategy] may have an effect because it means prioritizing competing demands [mechanism] to generate a positive implementation climate [proximal outcome]. Research questions that allow for specific mechanism-focused hypotheses have the potential to expedite the rate at which effective implementation strategies are identified.

#### Implementation theory

Ultimately, theory is necessary to drive hypotheses, explain implementation processes, and effectively inform implementation practice by providing guidance about when and in what contexts specific implementation strategies should or should not be used. Implementation theories can offer mechanisms that extend across levels of analysis (e.g., intrapersonal, interpersonal, organizational, community, macro policy [130]). However, there is a preponderance of frameworks and process models, with few theories in existence. Given that implementation is a process of behavior change at its core, in lieu of implementation-specific theories, many researchers draw upon classic theories from psychology, decision science, and organizational literatures, for instance. Because of this, the majority of the identified studies explored intrapersonal-level mechanisms, driven by their testing of social psychological theories such as the theory of planned behavior [65] and social cognitive theory [76, 77, 99]. Nine studies cited the diffusion of innovations [63, 64] as a theory guiding their mechanism investigation, which does extend beyond intrapersonal to emphasize interpersonal, and to some degree community level mechanisms, although we did not see this materialize in the included study analyses [63–65, 76, 77]. Moving forward, developing and testing theory is critical for advancing the study of implementation mechanisms because theories (implicitly or explicitly) tend to identify putative mechanisms instead of immutable determinants.

#### Measurement

Inadequate measurement has the potential to undermine our ability to advance this area of research. Our coding indicated that mechanisms were assessed almost exclusively via self-report (questionnaire, interview, focus group) suggesting that researchers conceptualize the diverse array of mechanisms to be latent constructs and not directly observable. This may indeed be appropriate, given that mechanisms are typically processes like learning and reflecting that occur within an individual and it is their proximal outcomes that are directly observable (e.g., knowledge acquisition, confidence, perceived control). However, conceptual, theoretical, and empirical work is needed to (a) articulate the theorized mechanisms for the 70+ strategies and proximal outcomes [128], (b) identify measures of implementation mechanisms and evaluate their psychometric evidence base [131] and pragmatic qualities [132], and (c) attempt to identify and rate or develop objective measures of proximal outcomes for use in real-time experimental manipulations of mechanism-outcome pairings.

#### Quantitative analytic approaches

The multilevel interrelations of factors implicated in an implementation process also call for sophisticated quantitative and qualitative methods to uncover mechanisms. With respect to quantitative methods, it was surprising that the Baron and Kenny [78] approach to mediation testing remains most prevalent despite that most studies are statistically underpowered to use this approach, and the other most common approach (i.e., the Sobel test [79]) relies on an assumption that the sampling distribution of the mediation effect is normal [14, 133], neither of which were reported on in any of the 12 included studies that used these methods. Williams [14] suggests the product of coefficients approach [134, 135] is more appropriate for mediation analysis because it is a highly general approach to both single and multi-level mediation models that minimizes type I error rates, maximizes statistical power, and enhances accuracy of confidence intervals [14]. The application of moderated mediation models and mediated moderator models will allow for a nuanced understanding of the complex interrelations among factors implicated in an implementation process.

#### Qualitative analytic approaches

Because this was the first review of implementation mechanisms across health disciplines, we believed it was important to be inclusive with respect to methods employed. Qualitative studies are important to advancing research on implementation mechanisms in part because they offer a data collection method in lieu of having an established measure to assess mechanisms quantitatively. Qualitative research is important for informing measure development work, but also for theory development given the richness of the data that can be gleaned. Qualitative inquiry can be more directive by developing hypotheses and generating interview guides to directly test mechanisms. Diagramming and tracing causal linkages can be informed by qualitative inquiry in a structured way that is explicit with regard to how the data informs our understanding of mechanisms. This kind of directed qualitative research is called for in the United Kingdom’s MRC Guidance for Process Evaluation [136]. We encourage researchers internationally to adopt this approach as it would importantly advance us beyond the descriptive studies that currently dominate the field.

### Limitations

There are several limitations to this study. First, we took an efficient approach to coding for study quality when applying the MMAT. Although it was a strength that we evaluated study quality, the majority of studies were assessed only by one research specialist. Second, we may have overlooked relevant process evaluations conducted in the UK where MRC Guidance stipulates inclusion of mechanisms that may have been described using terms not included in our search string. Third, although we identified several realist reviews, we did not include them in our systematic review because they conceptualize mechanisms differently than how they are treated in this review [137]. That is, realist synthesis posits that interventions are theories and that they imply specific mechanisms of action instead of separating mechanisms from the implementation strategies/interventions themselves [138]. Thus, including the realist operationalization would have further confused an already disharmonized literature with respect to mechanisms terminology but ultimately synthesizing findings from realist reviews with standard implementation mechanism evaluations will be important. Fourth, our characterization of the models tested in the identified studies may not reflect those intended by researchers given our attempt to offer conceptual consistency across studies, although we did reach out to corresponding authors for whom we wished to seek clarification on their study. Finally, because of the diversity of study designs and methods, and the inconsistent use of relevant terms, we are unable to synthesize across the studies and report on any robustly established mechanisms.

## Conclusion

This study represents the first systematic review of implementation mechanisms in health. Our inclusive approach yielded 46 qualitative, quantitative, and mixed methods studies, none of which met all seven criteria (i.e., strong association, specificity, consistency, experimental manipulation, timeline, gradient, plausibility or coherence) that are deemed critical for empirically establishing mechanisms. We found nine unique versions of models that attempted to uncover mechanisms, with only six exploring mediators of implementation strategies. The results of this review indicated inconsistent use of relevant terms (e.g., mechanisms, determinants) for which we offer guidance to achieve precision and encourage greater specificity in articulating research questions and hypotheses that allow for careful testing of causal relations among variables of interest. Implementation science will benefit from both quantitative and qualitative research that is more explicit in their attempt to uncover mechanisms. In doing so, our research will allow us to test the idea that more is better and move toward parsimony both for standardized and tailored approaches to implementation.

## Supplementary information

**Additional file 1: Figure S1.** Inclusion and Exclusion Criteria and Definitions.

**Additional file 2.** PRISMA 2009 Checklist.

**Additional file 3. ** Emergent Mechanism Models.

## Abbreviations

EBPs: Evidence-based practice
MMAT: Mixed methods appraisal tool
PARiHS: Promoting Action on Research in Health Services
HLM: Hierarchical linear modeling
SEM: Structural equation modeling

## Footnotes

1 A mediator can point toward a mechanism as it is an intervening variable that may account (statistically) for the relation between the independent variable (strategy) and the dependent variable (implementation outcome), revealing one possible causal pathway for the observed effect [4]. Compared to mediators, mechanisms are conceptualized as more precise in their description of the operations underlying causal processes [5].

2 Key differences in Williams’ [14] search method are important to note. Williams first conducted a broad search for randomized controlled trials concerning implementation or dissemination of evidence-based therapies. Only after screening references for these criteria, did Williams narrow the search to studies that specifically addressed mediators. Conversely, the present method included mediators/moderators/mechanisms as terms in the initial search string. Additionally, Williams hand searched references included in four previous reviews of implementation strategies in mental health.

3 We refer to variables in the ways the study authors did, even if we might have a different way in which we would approach their conceptualization.

## References

[1] Eccles MP, Mittman BS (2006). Welcome to implementation science. Implement Sci..

[2] Powell BJ, McMillen JC, Proctor EK, Carpenter CR, Griffey RT, Bunger AC (2012). A compilation of strategies for implementing clinical innovations in health and mental health. Med Care Res Rev..

[3] Powell BJ, Waltz TJ, Chinman MJ, Damschroder LJ, Smith JL, Matthieu MM (2015). A refined compilation of implementation strategies: results from the Expert Recommendations for Implementing Change (ERIC) project. Implement Sci..

[4] Kazdin AE (2007). Mediators and mechanisms of change in psychotherapy research. Annu Rev Clin Psychol..

[5] Kraemer HC, Wilson GT, Fairburn CG, Agras WS (2002). Mediators and moderators of treatment effects in randomized clinical trials. Arch Gen Psychiatry..

[6] Gerring J (2001). Social science methodology: a criterial framework.

[7] Frazier PA, Tix AP, Barron KE (2004). Testing moderator and mediator effects in counseling psychology research.

[8] Hill AB (1965). The Environment and Disease: Association or Causation?. Proc R Soc Med..

[9] Bosch M, van der Weijden T, Wensing M, Grol R (2007). Tailoring quality improvement interventions to identified barriers: a multiple case analysis. J Eval Clin Pract..

[10] Claridge JA, Fabian TC (2005). History and development of evidence-based medicine. World J Surg..

[11] Cook SC, Schwartz AC, Kaslow NJ (2017). Evidence-Based psychotherapy: advantages and challenges. Neurotherapeutics..

[12] Dissemination and Implementation Research in Health (R01 Clinical Trial Optional). National Institutes of Health (NIH); 2019. https://grants.nih.gov/grants/guide/pa-files/PAR-19-274.html.

[13] Thomas J, Brunton J, Graziosi S (2010). EPPI-Reviewer 4: software for research synthesis.

[14] Williams NJ (2016). Multilevel mechanisms of implementation strategies in mental health: integrating theory, research, and practice. Adm Policy Ment Health..

[15] Hong QN, Pluye P, Fabregues S, Bartlett G, Boardman F, Cargo M, et al. Mixed Methods Appraisal Tool (MMAT) Montreal, Canada: McGill University; 2018 [Available from: http://mixedmethodsappraisaltoolpublic.pbworks.com/w/file/fetch/127916259/MMAT_2018_criteria-manual_2018-08-01_ENG.pdf.

[16] Bardosh KL, Murray M, Khaemba AM, Smillie K, Lester R (2017). Operationalizing mHealth to improve patient care: a qualitative implementation science evaluation of the WelTel texting intervention in Canada and Kenya. Global Health..

[17] Brewster AL, Curry LA, Cherlin EJ, Talbert-Slagle K, Horwitz LI, Bradley EH (2015). Integrating new practices: a qualitative study of how hospital innovations become routine. Implement Sci..

[18] Carrera PM, Lambooij MS (2015). Implementation of out-of-office blood pressure monitoring in the netherlands: from clinical guidelines to patients’ adoption of innovation. Medicine..

[19] Frykman M, Hasson H, Muntlin Athlin Å, von Thiele Schwarz U (2014). Functions of behavior change interventions when implementing multi-professional teamwork at an emergency department: a comparative case study. BMC Health Serv Res..

[20] Wiener-Ogilvie S, Huby G, Pinnock H, Gillies J, Sheikh A (2008). Practice organisational characteristics can impact on compliance with the BTS/SIGN asthma guideline: qualitative comparative case study in primary care. BMC Fam Pract..

[21] Atkins MS, Frazier SL, Leathers SJ, Graczyk PA, Talbott E, Jakobsons L (2008). Teacher key opinion leaders and mental health consultation in low-income urban schools. J Consult Clin Psychol..

[22] Baer JS, Wells EA, Rosengren DB, Hartzler B, Beadnell B, Dunn C (2009). Agency context and tailored training in technology transfer: a pilot evaluation of motivational interviewing training for community counselors. J Subst Abuse Treat..

[23] Bonetti D, Eccles M, Johnston M, Steen N, Grimshaw J, Baker R (2005). Guiding the design and selection of interventions to influence the implementation of evidence-based practice: an experimental simulation of a complex intervention trial. Soc Sci Med..

[24] Garner BR, Godley SH, Bair CML (2011). The impact of pay-for-performance on therapists’ intentions to deliver high quality treatment. J Subst Abuse Treat..

[25] Glisson C, Schoenwald SK, Hemmelgarn A, Green P, Dukes D, Armstrong KS (2010). Randomized trial of MST and ARC in a two-level evidence-based treatment implementation strategy. J Consult Clin Psychol..

[26] Holth P, Torsheim T, Sheidow AJ, Ogden T, Henggeler SW (2011). Intensive quality assurance of therapist adherence to behavioral interventions for adolescent substance use problems. J Child Adolesc Subst Abuse..

[27] Lee H, Hall A, Nathan N, Reilly KL, Seward K, Williams CM (2018). Mechanisms of implementing public health interventions: a pooled causal mediation analysis of randomised trials. Implement Sci..

[28] Lochman JE, Boxmeyer C, Powell N, Qu L, Wells K, Windle M (2009). Dissemination of the coping power program: importance of intensity of counselor training. J Consult Clin Psychol..

[29] Rapkin BD, Weiss E, Lounsbury D, Michel T, Gordon A, Erb-Downward J (2017). Reducing Disparities in cancer screening and prevention through community-based participatory research partnerships with local libraries: a comprehensive dynamic trial. Am J Community Psychol..

[30] Rohrbach LA, Graham JW, Hansen WB (1993). Diffusion of a school-based substance abuse prevention program: predictors of program implementation. Prev Med..

[31] Seys D, Bruyneel L, Sermeus W, Lodewijckx C, Decramer M, Deneckere S (2018). Teamwork and adherence to recommendations explain the effect of a care pathway on reduced 30-day readmission for patients with a COPD exacerbation. COPD..

[32] Williams NJG, C. The role of organizational culture and climate in the dissemination and implementation of empirically-supported treatments for youth. Dissemination and implementation of evidence based practices in child and adolescent mental health. New York: Oxford University Press; 2014. p. 61-81.

[33] Williams NJ, Glisson C, Hemmelgarn A, Green P (2017). Mechanisms of change in the ARC Organizational strategy: increasing mental health clinicians' EBP adoption through improved organizational culture and capacity. Adm Policy Ment Health..

[34] Aarons GA, Sommerfeld DH, Walrath-Greene CM (2009). Evidence-based practice implementation: the impact of public versus private sector organization type on organizational support, provider attitudes, and adoption of evidence-based practice. Implement Sci..

[35] Becker SJ, Squires DD, Strong DR, Barnett NP, Monti PM, Petry NM (2016). Training opioid addiction treatment providers to adopt contingency management: a prospective pilot trial of a comprehensive implementation science approach. Subst Abus..

[36] Beenstock J, Sniehotta FF, White M, Bell R, Milne EMG, Araujo-Soares V (2012). What helps and hinders midwives in engaging with pregnant women about stopping smoking?. A cross-sectional survey of perceived implementation difficulties among midwives in the North East of England. Implement Sci..

[37] Beets MW, Flay BR, Vuchinich S, Acock AC, Li KK, Allred C (2008). School climate and teachers' beliefs and attitudes associated with implementation of the positive action program: a diffusion of innovations model. Prev Sci..

[38] Bonetti D, Johnston M, Clarkson J, Turner S (2009). Applying multiple models to predict clinicians' behavioural intention and objective behaviour when managing children's teeth. Psychol Health..

[39] Chou AF, Vaughn TE, McCoy KD, Doebbeling BN (2011). Implementation of evidence-based practices: applying a goal commitment framework. Health Care Manage Rev..

[40] Chambers D, Simpson L, Neta G, UvT S, Percy-Laurry A, Aarons GA (2017). Proceedings from the 9th annual conference on the science of dissemination and implementation. Implementation Sci.

[41] David P, Schiff M (2017). Self-efficacy as a mediator in bottom-up dissemination of a Research-supported intervention for young, traumatized children and their families. J Evid Inf Soc Work..

[42] Edmunds JM, Read KL, Ringle VA, Brodman DM, Kendall PC, Beidas RS. Sustaining clinician penetration, attitudes and knowledge in cognitive-behavioral therapy for youth anxiety. Implement Sci. 2014;9. 10.1186/s13012-014-0089-9 PMC4223397 25030651

[43] Gnich W, Sherriff A, Bonetti D, Conway DI, Macpherson LMD (2018). The effect of introducing a financial incentive to promote application of fluoride varnish in dental practice in Scotland: a natural experiment. Implement Sci..

[44] Guerrero EG, Frimpong J, Kong Y, Fenwick K (2018). Aarons GA.

[45] Huis A, Holleman G, van Achterberg T, Grol R, Schoonhoven L, Hulscher M (2013). Explaining the effects of two different strategies for promoting hand hygiene in hospital nurses: a process evaluation alongside a cluster randomised controlled trial. Implement Sci..

[46] Little MA, Pokhrel P, Sussman S, Rohrbach LA (2015). The process of adoption of evidence-based tobacco use prevention programs in California schools. Prev Sci..

[47] Llasus L, Angosta AD, Clark M (2014). Graduating baccalaureate students' evidence-based practice knowledge, readiness, and implementation. J Nurs Educ..

[48] Nelson TD, Steele RG (2007). Predictors of practitioner self-reported use of evidence-based practices: practitioner training, clinical setting, and attitudes toward research. Adm Policy Ment Health..

[49] Potthoff S, Presseau J, Sniehotta FF, Johnston M, Elovainio M, Avery L (2017). Planning to be routine: habit as a mediator of the planning-behaviour relationship in healthcare professionals. Implement Sci..

[50] Presseau J, Grimshaw JM, Tetroe JM, Eccles MP, Francis JJ, Godin G (2016). A theory-based process evaluation alongside a randomised controlled trial of printed educational messages to increase primary care physicians' prescription of thiazide diuretics for hypertension [ISRCTN72772651]. Implement Sci..

[51] Simmonds MJ, Derghazarian T, Vlaeyen JW (2012). Physiotherapists' knowledge, attitudes, and intolerance of uncertainty influence decision making in low back pain. Clin J Pain..

[52] Stockdale SE, Rose D, Darling JE, Meredith LS, Helfrich CD, Dresselhaus TR (2018). Communication among team members within the patient-centered medical home and patient satisfaction with providers: the mediating role of patient-provider communication. Med Care..

[53] Wanless SB, Rimm-Kaufman SE, Abry T, Larsen RA, Patton CL (2015). Engagement in training as a mechanism to understanding fidelity of implementation of the responsive classroom approach. Prev Sci..

[54] Armson H, Roder S, Elmslie T, Khan S, Straus SE (2018). How do clinicians use implementation tools to apply breast cancer screening guidelines to practice?. Implement Sci..

[55] Birken SA, Lee S-YD, Weiner BJ, Chin MH, Chiu M, Schaefer CT (2015). From strategy to action: how top managers’ support increases middle managers’ commitment to innovation implementation in healthcare organizations. Health Care Manage Rev..

[56] Kauth MR, Sullivan G, Blevins D, Cully JA, Landes RD, Said Q (2010). Employing external facilitation to implement cognitive behavioral therapy in VA clinics: a pilot study. Implement Sci..

[57] Lukas CV, Mohr DC, Meterko M (2009). Team effectiveness and organizational context in the implementation of a clinical innovation. Qual Manag Health Care..

[58] Panzano PC, Sweeney HA, Seffrin B, Massatti R, Knudsen KJ (2012). The assimilation of evidence-based healthcare innovations: a management-based perspective. J Behav Health Serv Res..

[59] Rangachari P, Madaio M, Rethemeyer RK, Wagner P, Hall L, Roy S (2015). The evolution of knowledge exchanges enabling successful practice change in two intensive care units. Health Care Manage Rev..

[60] Shrubsole K, Worrall L, Power E, O'Connor DA (2018). The acute aphasia implementation study (AAIMS): a pilot cluster randomized controlled trial. Int J Lang Commun Disord..

[61] Scott SD, Albrecht L, O'Leary K, Ball GD, Hartling L, Hofmeyer A (2012). Systematic review of knowledge translation strategies in the allied health professions. Implement Sci..

[62] Yamada J, Squires JE, Estabrooks CA, Victor C, Stevens B, Pain CTiCs (2017). The role of organizational context in moderating the effect of research use on pain outcomes in hospitalized children: a cross sectional study. BMC Health Serv Res.

[63] Rogers E (1995). Diffusion of innovations.

[64] Rogers E (1983). Diffusion of Innovations.

[65] Ajzen I (1991). The theory of planned behavior. Organ Behav Hum Decis Process..

[66] Michie S, Johnston M, Abraham C, Lawton R, Parker D, Walker A (2005). Making psychological theory useful for implementing evidence based practice: a consensus approach. Qual Saf Health Care..

[67] Kitson A, Harvey G, McCormack B (1998). Enabling the implementation of evidence based practice: a conceptual framework. Qual Health Care..

[68] Damschroder LJ, Aron DC, Keith RE, Kirsh SR, Alexander JA, Lowery JC (2009). Fostering implementation of health services research findings into practice: a consolidated framework for advancing implementation science. Implement Sci..

[69] Klein KJ, Sorra JS (1996). The challenge of innovation implementation. Acad Manage Rev..

[70] Davis FD (1989). Perceived usefulness, perceived ease of use, and user acceptance of information technology. MIS Quarterly..

[71] Thompson RS, Higgins CA, Howell JM (1991). Personal computing: toward a conceptual model of utilization. MIS Quarterly..

[72] Braksick LW (2007). Unlock behavior, unleash profits: developing leadership behavior that drives profitability in your organization.

[73] Johnson J, Dakens L, Edwards P, Morse N (2008). SwitchPoints: culture change on the fast track to business success.

[74] Hedeker D, Gibbons RD (2006). Longitudinal data analysis.

[75] Krull JL, MacKinnon DP (2001). Multilevel modeling of individual and group level mediated effects. Multivariate Behav Res..

[76] Bandura A (1997). Self-efficacy: the exercise of control.

[77] Bandura A (2000). Exercise of human agency through collective efficacy. Curr Dir Psychol Sci..

[78] Baron RM, Kenny DA (1986). The moderator-mediator variable distinction in social psychological research: conceptual, strategic, and statistical considerations. J Pers Soc Psychol..

[79] Sobel ME, Leinhart S (1982). Asymptotic confidence intervals for indirect effects in structural equation models. Sociological Methodology.

[80] Raudenbush SW, Bryk AS, Cheong YF, Congdon RT (2004). HLM7: hierarchical linear and nonlinear modeling.

[81] Hosmer DW, Lemeshow S (1989). Applied logistic regression.

[82] Raudenbush SW, Bryk A, Congdon RT (2005). HLM 6.

[83] Singer JD, Willet JB (2003). Applied longitudinal data analysis: modeling change and event occurrence.

[84] Cane J, O'Connor D, Michie S (2012). Validation of the theoretical domains framework for use in behaviour change and implementation research. Implement Sci..

[85] Imai K, Keele L, Tingley D (2010). A general approach to causal mediation analysis. Psychol Methods..

[86] van Buuren SG-O, K. Mice: multivariate imputation by chained equations in R. J Stat Softw. 2010:1–68.

[87] Rogers EM (2003). Diffusion of innovations.

[88] Raudenbush SW, Liu X (2000). Statistical power and optimal design for multisite randomized trials. Psychol Methods..

[89] Allison PD (1984). Event history analysis.

[90] Yuk Fai C, Randall PF, Stephen WR (2001). Efficiency and robustness of alternative estimators for two- and three-level models: the case of NAEP. J Educ Behav Stat..

[91] Hox JJ, Maas CJM (2001). The accuracy of multilevel structural equation modeling with pseudobalanced groups and small samples. Struct Equ Model..

[92] Zhang Z, Zyphur MJ, Preacher KJ (2009). Testing multilevel mediation using hierarchical linear models: problems and solutions. Organizational Research Methods..

[93] Scott WR (2001). Institutions and Organizations.

[94] Eisenberger R, Huntington R, Hutchison S, Sowa D (1986). Perceived organizational support. Journal of Applied Psychology..

[95] Preacher KJ, Hayes AF (2004). SPSS and SAS procedures for estimating indirect effects in simple mediation models. Behav Res Methods Instrum Comput..

[96] Chen HT, Reynolds HJ, Walber HJ (1998). Theory-driven evaluations. Advances in educational productivity: evaluation research for educational productivity.

[97] Marsh HW, Hau KT, Balla JR, Grayson D (1998). Is More Ever Too Much? The Number of indicators per factor in confirmatory factor analysis. Multivariate Behav Res..

[98] Bandalos DL, Finney SJ, Marcoulides GA (2001). Item parceling issues in structural equation modeling. New developments and techniques in structural equation modeling.

[99] Bandura A (1998). Health promotion from the perspective of social cognitive theory. Psychol Health..

[100] Blackman D (1974). Operant conditioning: an experimental analysis of behaviour.

[101] Gollwitzer PM (1999). Implementation intentions: strong effects of simple plans. Am Psychol.

[102] Leventhal H, Nerenz D, Steele DJ, Baum A, Taylor SE, Singer JE (1984). Illness representations and coping with health threats. Handbook of psychology and health, volume 4: social psychological aspects of health.

[103] Weinstein N (1988). The precaution adoption process. Health Psychol..

[104] Prochaska JO, DiClemente CC (1983). Stages and processes of self-change of smoking: toward an integrative model of change. J Consult Clin Psychol..

[105] Landy FJ, Becker W, Cumming LL, Staw BM (1987). Motivation theory reconsidered. Research in organizational behavior.

[106] Locke EA, Latham GP (2002). Building a practically useful theory of goal setting and task motivation: a 35-year odyssey. Am Psychol..

[107] Kennedy P (2003). A guide to econometrics.

[108] Joreskog KGS, D. (1996). LISRELR 8: User’s reference guide.

[109] Valente TW (1996). Social network thresholds in the diffusion of innovations. Social Networks..

[110] Hayes AF (2009). Beyond Baron and Kenny: Statistical mediation analysis in the new millennium. Communication Monographs..

[111] Aarons GA, Hurlburt M, Horwitz SM (2011). Advancing a conceptual model of evidence-based practice implementation in public service sectors. Adm Policy Ment Health..

[112] Raudenbush SW, Bryk AS (2002). Hierarchical linear models.

[113] Bryk AS, Raudenbush SW (1992). Hierarchical linear models.

[114] Muthén LK, Muthén BO. Mplus user's guide Los Angeles, CA: Muthén & Muthén 2012 [Seventh Edition:[Available from: https://www.statmodel.com/download/usersguide/Mplus%20user%20guide%20Ver_7_r3_web.pdf.

[115] Bentler PM (2007). On tests and indices for evaluating structural models. Personal Individ Differ..

[116] MacKinnon DP, Fairchild AJ, Fritz MS (2007). Mediation analysis. Annu Rev Psychol..

[117] Graham I, Logan J, Harrison M, Straus S, Tetroe J, Caswell W, et al. Lost in knowledge translation: time for a map? J Contin Educ Health Prof. 2006;26. 10.1002/chp.47 16557505

[118] Epstein S, Pervin LA (1990). Cognitive-experiential self-theory. Handbook of personality: theory and research.

[119] Karlson KB, Holm A, Breen R (2012). Comparing Regression coefficients between same-sample Nested models using logit and probit: a new method. Sociological Methodology..

[120] Rycroft-Malone J, Kitson A, Harvey G, McCormack B, Seers K, Titchen A (2002). Ingredients for change: revisiting a conceptual framework. BMJ Qual Saf..

[121] Yukl G, Gordon A, Taber T (2002). A hierarchical taxonomy of leadership behavior: integrating a half century of behavior research. J Leadersh Organ Stud..

[122] Shrout PE, Bolger N (2002). Mediation in experimental and nonexperimental studies: new procedures and recommendations. Psychol Methods..

[123] Fixsen DL, Naoom SF, Blase KA, Friedman RM (2005). Implementation research: a synthesis of the literature.

[124] Frambach R (1993). An integrated model of organizational adoption and diffusion of innovations. Eur J Mark..

[125] Institute of Medicine (IOM). Crossing the quality chasm: a new health system for the 21st century. Washington, DC: Institute of Medicine, National Academy Press; 2001.

[126] Preacher KJ, Hayes AF (2008). Asymptotic and resampling strategies for assessing and comparing indirect effects in multiple mediator models. Behav Res Methods..

[127] Stahmer AC, Suhrheinrich J, Schetter PL, McGee HE (2018). Exploring multi-level system factors facilitating educator training and implementation of evidence-based practices (EBP): a study protocol. Implement Sci..

[128] Lewis CC, Klasnja P, Powell BJ, Lyon AR, Tuzzio L, Jones S (2018). From classification to causality: advancing understanding of mechanisms of change in implementation science. Front Public Health..

[129] Proctor E, Silmere H, Raghavan R, Hovmand P, Aarons G, Bunger A (2011). Outcomes for implementation research: conceptual distinctions, measurement challenges, and research agenda. Adm Policy Ment Health..

[130] Weiner BJ, Lewis MA, Clauser SB, Stitzenberg KB (2012). In search of synergy: strategies for combining interventions at multiple levels. J Natl Cancer Inst Monogr..

[131] Lewis CC, Weiner BJ, Stanick C, Fischer SM (2015). Advancing implementation science through measure development and evaluation: a study protocol. Implement Sci..

[132] Powell BJ, Stanick CF, Halko HM, Dorsey CN, Weiner BJ, Barwick MA (2017). Toward criteria for pragmatic measurement in implementation research and practice: a stakeholder-driven approach using concept mapping. Implement Sci..

[133] Wu AD, Zumbo BD (2007). Understanding and using mediators and moderators. Soc Indic Res..

[134] MacKinnon DP, Lockwood CM, Hoffman JM, West SG, Sheets V (2002). A comparison of methods to test mediation and other intervening variable effects. Psychol Methods..

[135] Pituch KA, Murphy DL, Tate RL (2009). Three-level models for indirect effects in school- and class-randomized experiments in education. J Exp Educ..

[136] Moore GF, Audrey S, Barker M, Bond L, Bonell C, Hardeman W (2015). Process evaluation of complex interventions: Medical Research Council guidance. BMJ : British Medical Journal..

[137] Pawson R, Manzano-Santaella A (2012). A realist diagnostic workshop. Evaluation..

[138] Pawson R, Greenhalgh T, Harvey G, Walshe K (2004). Realist synthesis: an introduction.

