# Supplementary material for: A systematic review of empirical studies examining mechanisms of implementation in health
Source: Implement Sci. 2020 Apr 16;15:21. doi: 10.1186/s13012-020-00983-3 (PMC7164241; doi:10.1186/s13012-020-00983-3)
Supplement: Supplementary file 1 — Additional file 1: Figure S1. Inclusion and Exclusion Criteria and Definitions. [file 13012_2020_983_MOESM1_ESM.docx]

| **Additional File 1:** *Inclusion and Exclusion Criteria and Definitions* | |
| --- | --- |
| **Criteria** | **Definition** |
| Exclude on “mechanism” used in a different context | This code was applied when usage of the term “mechanism” implied “a process, technique or system for achieving results” or when the term “mechanism” was used in a context other than Implementation Science (e.g. mechanism of treatment effect, mechanism of injury, mechanism of drug effectiveness) |
| Exclude on “mediation” used in a different context | This code was applied when usage of the term “mechanism” implied conflict mediation/mediator or when the term “mediation” was used in a context other than Implementation Science |
| Exclude on “moderator” used in a different context | This code was applied when usage of the term implied a small amount (e.g. the intervention was moderately effective), or when the term was used in a context of than implementation science (e.g. sex was found to be a moderator of treatment response) |
| Exclude on concept paper/opinion piece | This code was applied when a paper did not test a mechanism, rather presented a view point or concept. Examples include articles that presented: debates, methodologies, framework, point of view or guidelines. |
| Exclude on review paper | This code was applied when a paper did not test a mechanism, rather it reviews the literature (could be a systematic, scoping or unspecified review), the abstract must explicitly state that the aim of the paper was to conduct a review |
| Exclude not Implementation Science | This code was applied when a paper was not related to implementation science per the definition provided in the text. Papers that addressed any stage of implementation of the EPIS model (Exploration, Preparation, Implementation, Sustainment) were eligible for inclusion. |
| Exclude on future direction | This code was applied when mechanisms, mediators, or moderators were only suggested as topics of future study, rather than explicitly examined in the current study. |
| Exclude on realist evaluation | This code was applied when the study utilized realist evaluation methodology to examine mechanisms. Mechanisms are defined in realist evaluations in a different manner than the definition applied in the current review. |
