# Supplementary material for: A systematic review of empirical studies examining mechanisms of implementation in health
Source: Implement Sci. 2020 Apr 16;15:21. doi: 10.1186/s13012-020-00983-3 (PMC7164241; doi:10.1186/s13012-020-00983-3)
Supplement: Supplementary file 3 — Additional file 3. Emergent Mechanism Models. [file 13012_2020_983_MOESM3_ESM.pptx]

## Slide 1
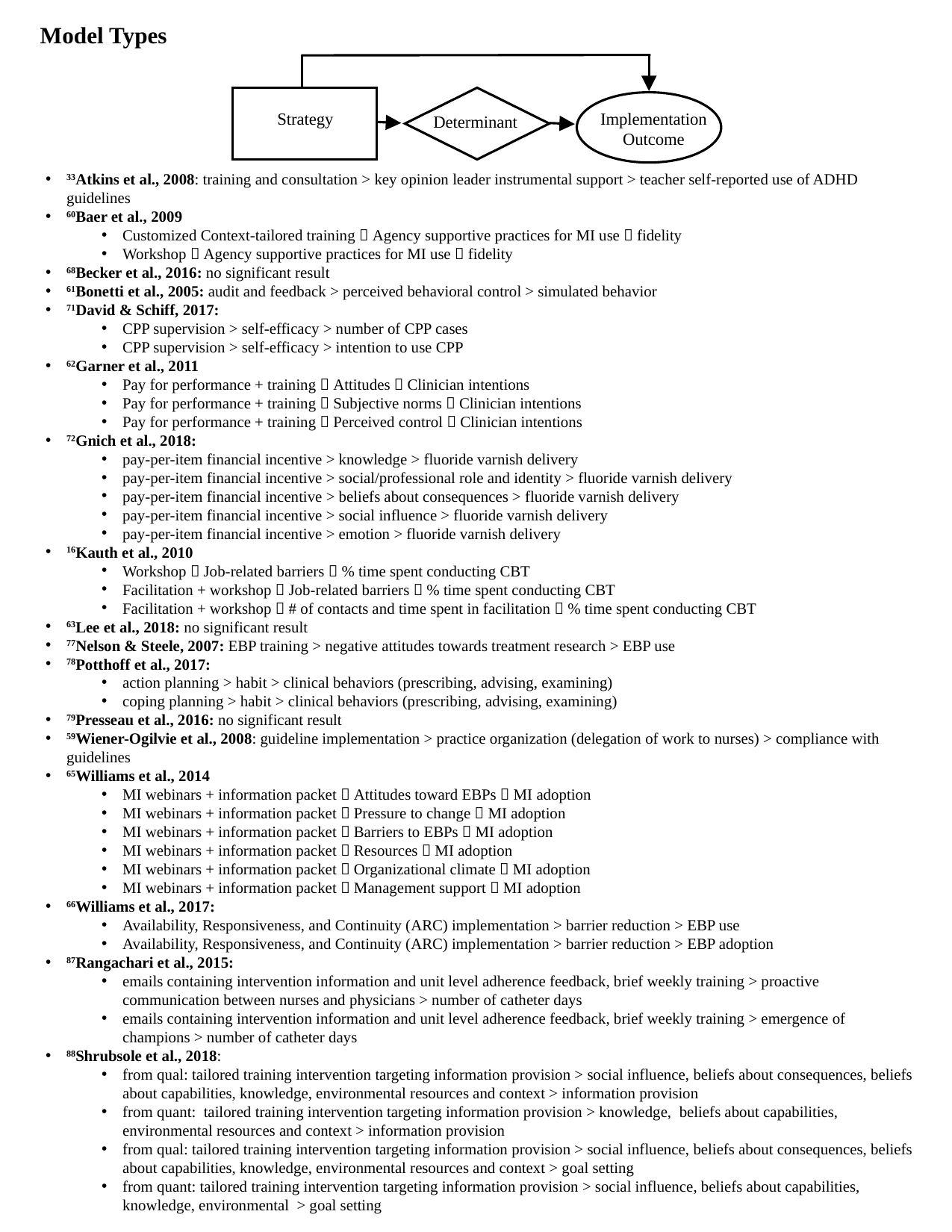

Model Types
Strategy
Implementation Outcome
Determinant
33Atkins et al., 2008: training and consultation > key opinion leader instrumental support > teacher self-reported use of ADHD guidelines
60Baer et al., 2009
Customized Context-tailored training  Agency supportive practices for MI use  fidelity
Workshop  Agency supportive practices for MI use  fidelity
68Becker et al., 2016: no significant result
61Bonetti et al., 2005: audit and feedback > perceived behavioral control > simulated behavior
71David & Schiff, 2017:
CPP supervision > self-efficacy > number of CPP cases
CPP supervision > self-efficacy > intention to use CPP
62Garner et al., 2011
Pay for performance + training  Attitudes  Clinician intentions
Pay for performance + training  Subjective norms  Clinician intentions
Pay for performance + training  Perceived control  Clinician intentions
72Gnich et al., 2018:
pay-per-item financial incentive > knowledge > fluoride varnish delivery
pay-per-item financial incentive > social/professional role and identity > fluoride varnish delivery
pay-per-item financial incentive > beliefs about consequences > fluoride varnish delivery
pay-per-item financial incentive > social influence > fluoride varnish delivery
pay-per-item financial incentive > emotion > fluoride varnish delivery
16Kauth et al., 2010
Workshop  Job-related barriers  % time spent conducting CBT
Facilitation + workshop  Job-related barriers  % time spent conducting CBT
Facilitation + workshop  # of contacts and time spent in facilitation  % time spent conducting CBT
63Lee et al., 2018: no significant result
77Nelson & Steele, 2007: EBP training > negative attitudes towards treatment research > EBP use
78Potthoff et al., 2017:
action planning > habit > clinical behaviors (prescribing, advising, examining)
coping planning > habit > clinical behaviors (prescribing, advising, examining)
79Presseau et al., 2016: no significant result
59Wiener-Ogilvie et al., 2008: guideline implementation > practice organization (delegation of work to nurses) > compliance with guidelines
65Williams et al., 2014
MI webinars + information packet  Attitudes toward EBPs  MI adoption
MI webinars + information packet  Pressure to change  MI adoption
MI webinars + information packet  Barriers to EBPs  MI adoption
MI webinars + information packet  Resources  MI adoption
MI webinars + information packet  Organizational climate  MI adoption
MI webinars + information packet  Management support  MI adoption
66Williams et al., 2017:
Availability, Responsiveness, and Continuity (ARC) implementation > barrier reduction > EBP use
Availability, Responsiveness, and Continuity (ARC) implementation > barrier reduction > EBP adoption
87Rangachari et al., 2015:
emails containing intervention information and unit level adherence feedback, brief weekly training > proactive communication between nurses and physicians > number of catheter days
emails containing intervention information and unit level adherence feedback, brief weekly training > emergence of champions > number of catheter days
88Shrubsole et al., 2018:
from qual: tailored training intervention targeting information provision > social influence, beliefs about consequences, beliefs about capabilities, knowledge, environmental resources and context > information provision
from quant: tailored training intervention targeting information provision > knowledge, beliefs about capabilities, environmental resources and context > information provision
from qual: tailored training intervention targeting information provision > social influence, beliefs about consequences, beliefs about capabilities, knowledge, environmental resources and context > goal setting
from quant: tailored training intervention targeting information provision > social influence, beliefs about capabilities, knowledge, environmental > goal setting

## Slide 2
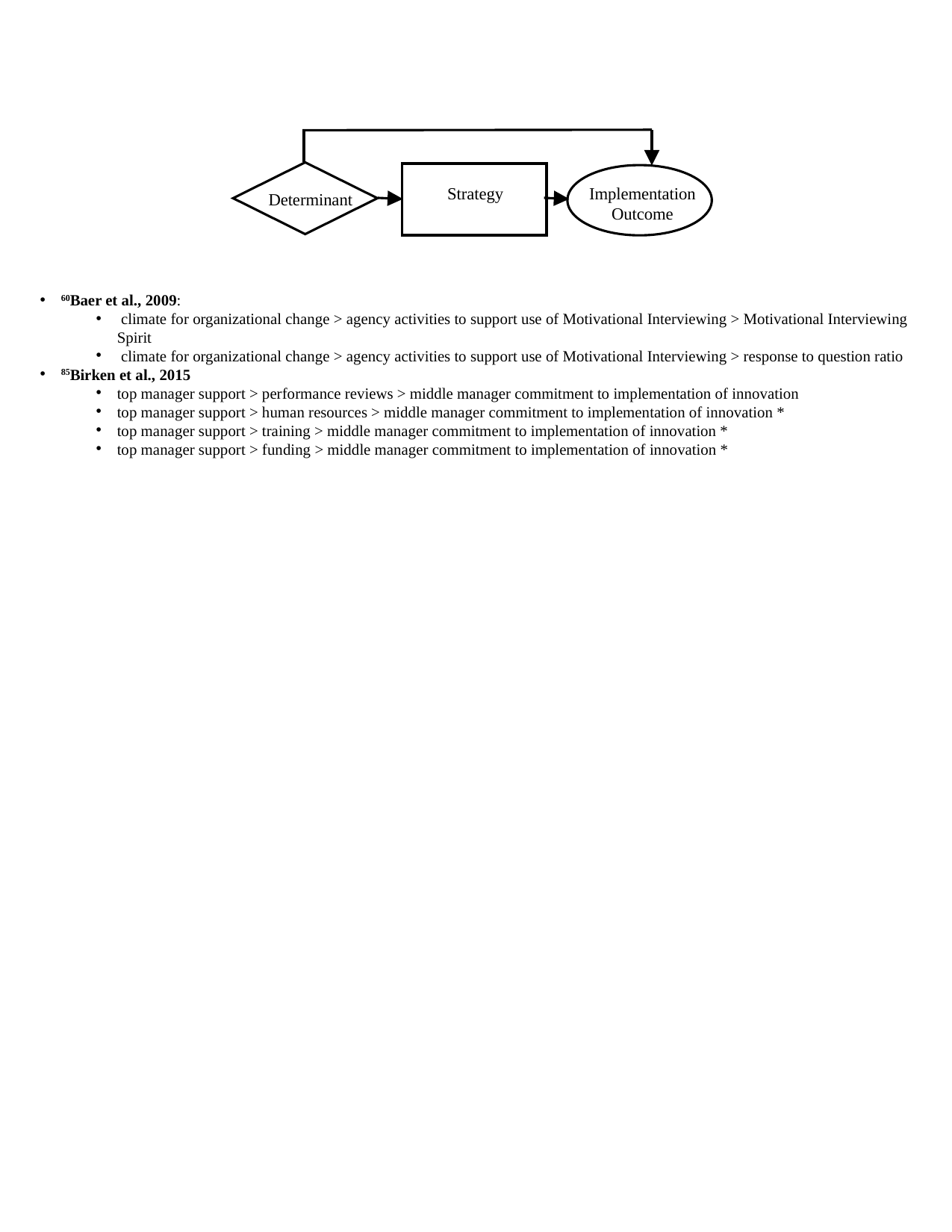

Strategy
Implementation Outcome
Determinant
60Baer et al., 2009:
 climate for organizational change > agency activities to support use of Motivational Interviewing > Motivational Interviewing Spirit
 climate for organizational change > agency activities to support use of Motivational Interviewing > response to question ratio
85Birken et al., 2015
top manager support > performance reviews > middle manager commitment to implementation of innovation
top manager support > human resources > middle manager commitment to implementation of innovation *
top manager support > training > middle manager commitment to implementation of innovation *
top manager support > funding > middle manager commitment to implementation of innovation *

## Slide 3
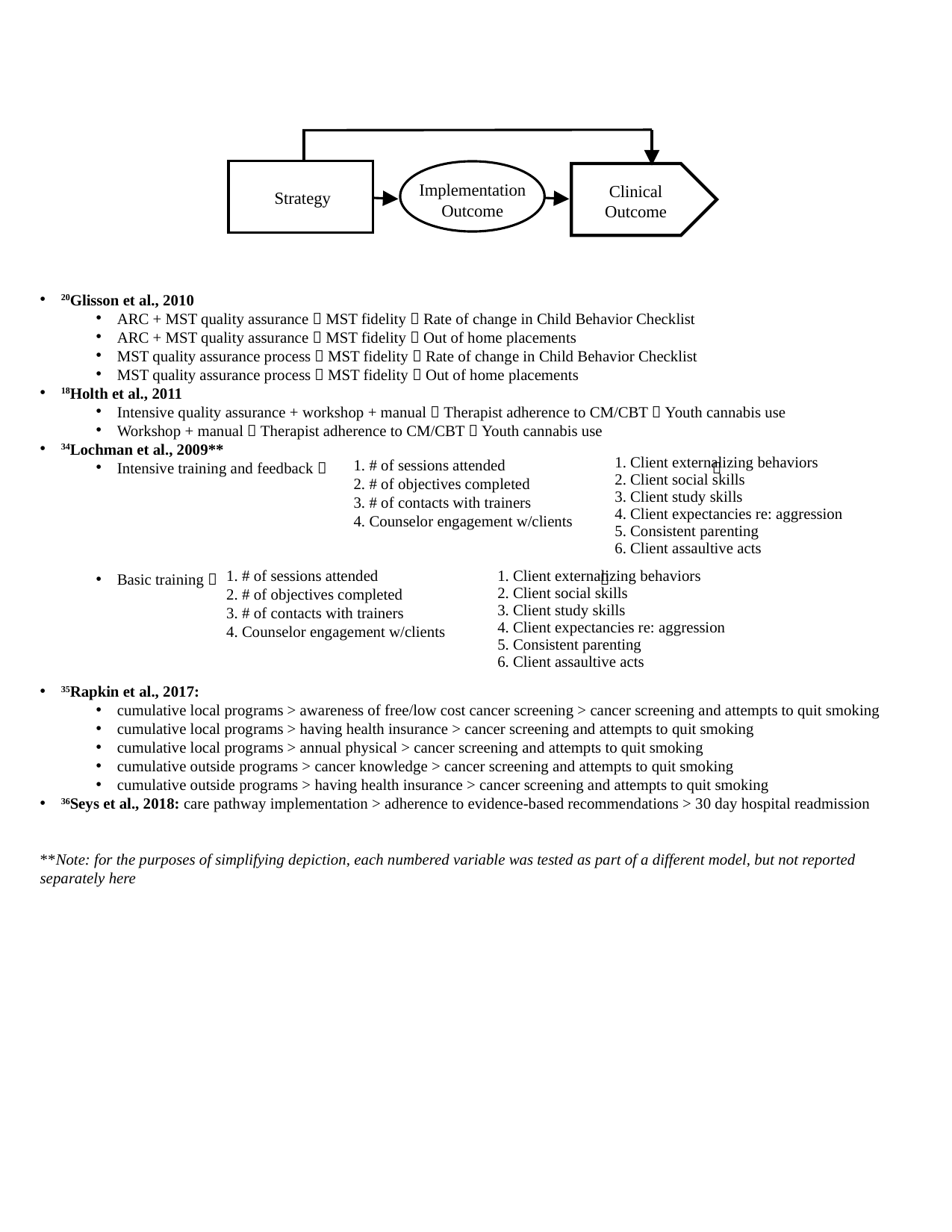

Implementation Outcome
Clinical Outcome
Strategy
20Glisson et al., 2010
ARC + MST quality assurance  MST fidelity  Rate of change in Child Behavior Checklist
ARC + MST quality assurance  MST fidelity  Out of home placements
MST quality assurance process  MST fidelity  Rate of change in Child Behavior Checklist
MST quality assurance process  MST fidelity  Out of home placements
18Holth et al., 2011
Intensive quality assurance + workshop + manual  Therapist adherence to CM/CBT  Youth cannabis use
Workshop + manual  Therapist adherence to CM/CBT  Youth cannabis use
34Lochman et al., 2009**
Intensive training and feedback  				 
Basic training  				 
35Rapkin et al., 2017:
cumulative local programs > awareness of free/low cost cancer screening > cancer screening and attempts to quit smoking
cumulative local programs > having health insurance > cancer screening and attempts to quit smoking
cumulative local programs > annual physical > cancer screening and attempts to quit smoking
cumulative outside programs > cancer knowledge > cancer screening and attempts to quit smoking
cumulative outside programs > having health insurance > cancer screening and attempts to quit smoking
36Seys et al., 2018: care pathway implementation > adherence to evidence-based recommendations > 30 day hospital readmission
**Note: for the purposes of simplifying depiction, each numbered variable was tested as part of a different model, but not reported separately here
| 1. Client externalizing behaviors 2. Client social skills 3. Client study skills 4. Client expectancies re: aggression 5. Consistent parenting 6. Client assaultive acts |
| --- |
| 1. # of sessions attended |
| --- |
| 2. # of objectives completed |
| 3. # of contacts with trainers |
| 4. Counselor engagement w/clients |
| 1. # of sessions attended |
| --- |
| 2. # of objectives completed |
| 3. # of contacts with trainers |
| 4. Counselor engagement w/clients |
| 1. Client externalizing behaviors 2. Client social skills 3. Client study skills 4. Client expectancies re: aggression 5. Consistent parenting 6. Client assaultive acts |
| --- |

## Slide 4
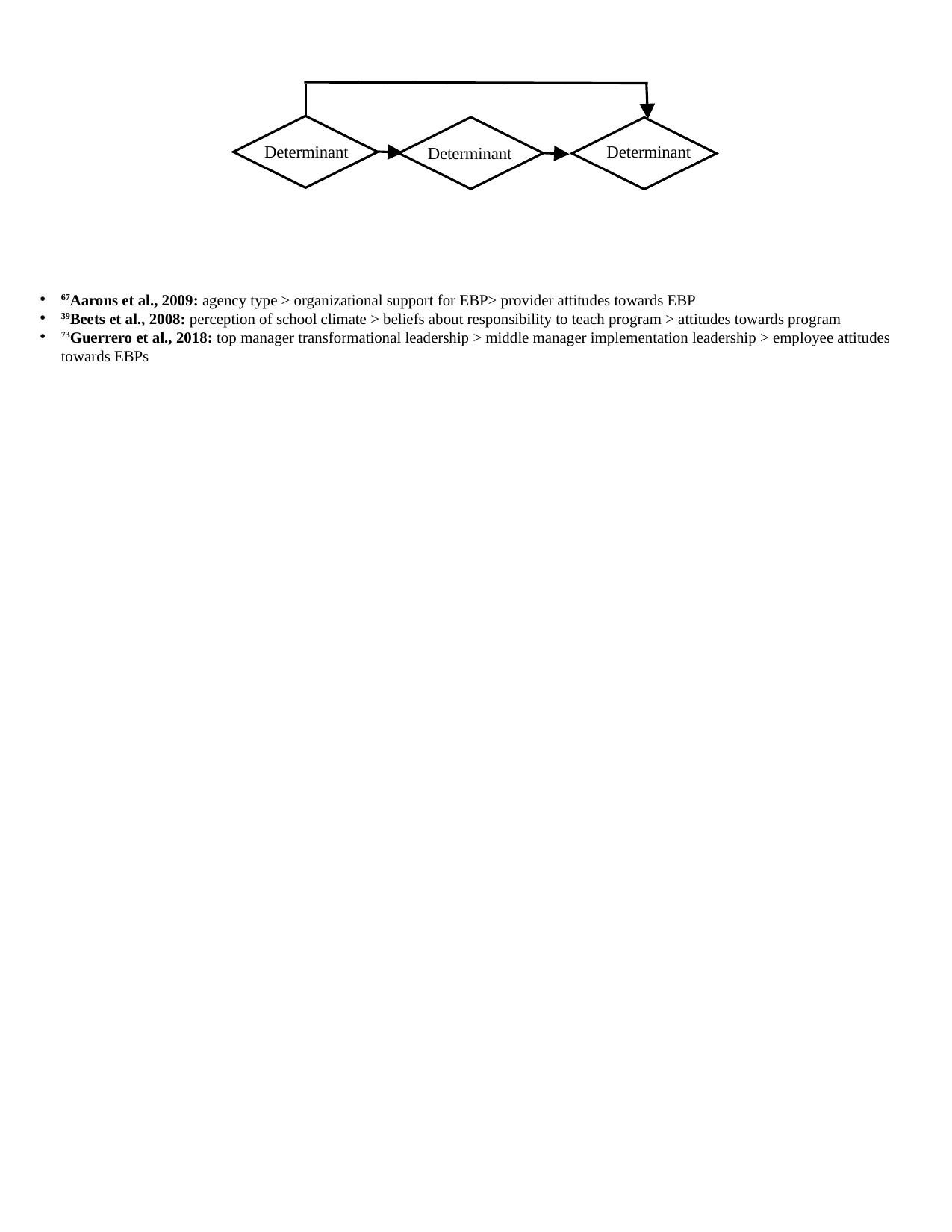

Determinant
Determinant
Determinant
67Aarons et al., 2009: agency type > organizational support for EBP> provider attitudes towards EBP
39Beets et al., 2008: perception of school climate > beliefs about responsibility to teach program > attitudes towards program
73Guerrero et al., 2018: top manager transformational leadership > middle manager implementation leadership > employee attitudes towards EBPs

## Slide 5
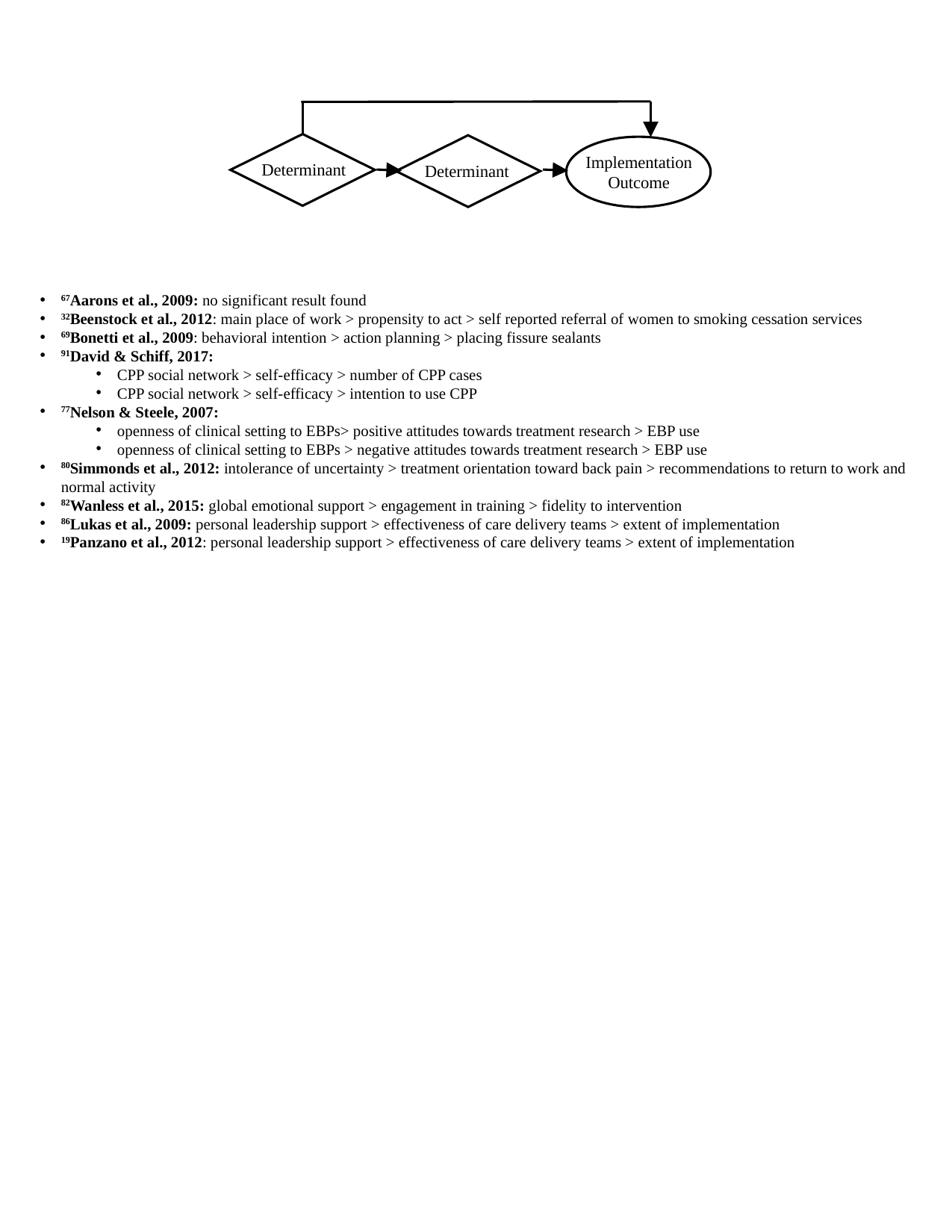

Implementation Outcome
Determinant
Determinant
67Aarons et al., 2009: no significant result found
32Beenstock et al., 2012: main place of work > propensity to act > self reported referral of women to smoking cessation services
69Bonetti et al., 2009: behavioral intention > action planning > placing fissure sealants
91David & Schiff, 2017:
CPP social network > self-efficacy > number of CPP cases
CPP social network > self-efficacy > intention to use CPP
77Nelson & Steele, 2007:
openness of clinical setting to EBPs> positive attitudes towards treatment research > EBP use
openness of clinical setting to EBPs > negative attitudes towards treatment research > EBP use
80Simmonds et al., 2012: intolerance of uncertainty > treatment orientation toward back pain > recommendations to return to work and normal activity
82Wanless et al., 2015: global emotional support > engagement in training > fidelity to intervention
86Lukas et al., 2009: personal leadership support > effectiveness of care delivery teams > extent of implementation
19Panzano et al., 2012: personal leadership support > effectiveness of care delivery teams > extent of implementation

## Slide 6
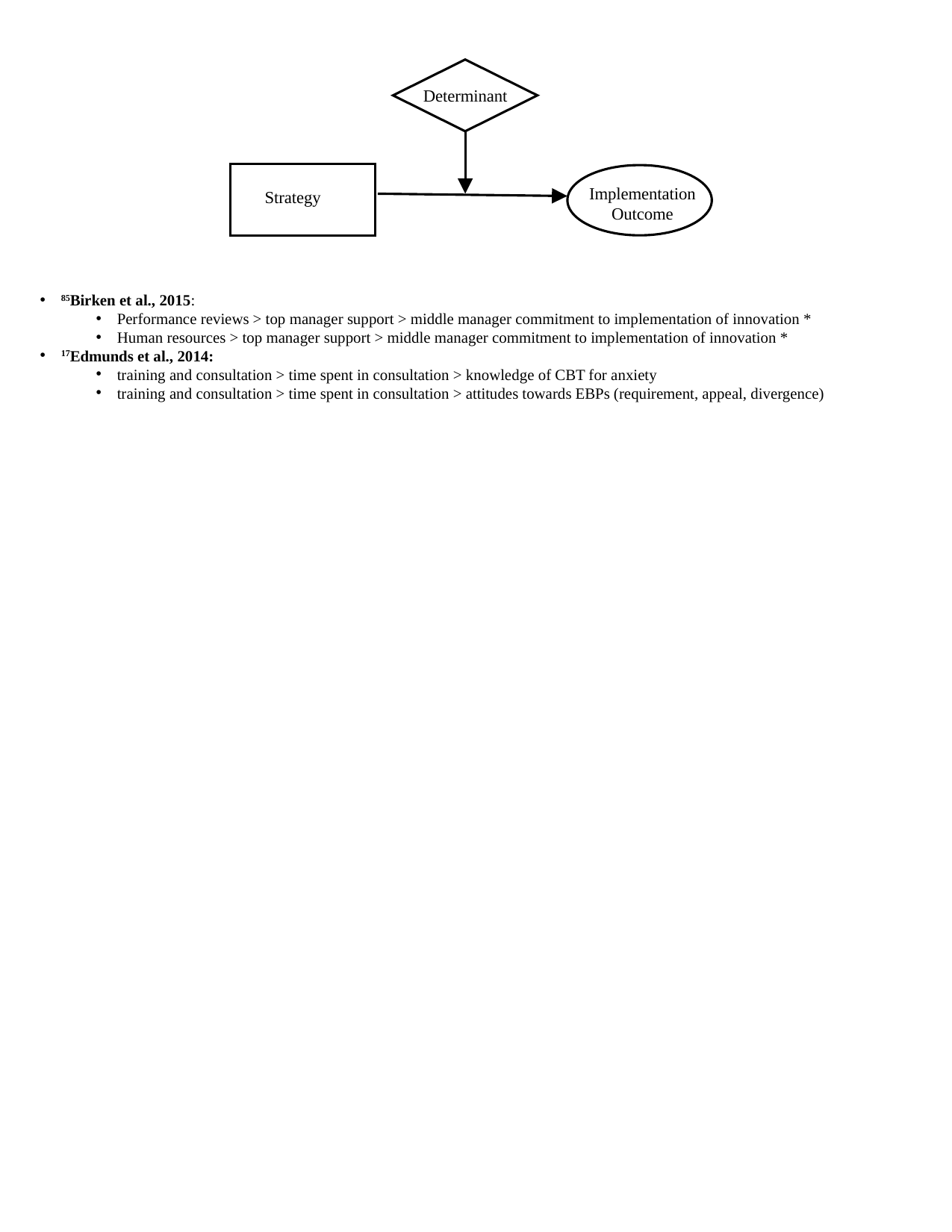

Determinant
Implementation Outcome
Strategy
85Birken et al., 2015:
Performance reviews > top manager support > middle manager commitment to implementation of innovation *
Human resources > top manager support > middle manager commitment to implementation of innovation *
17Edmunds et al., 2014:
training and consultation > time spent in consultation > knowledge of CBT for anxiety
training and consultation > time spent in consultation > attitudes towards EBPs (requirement, appeal, divergence)

## Slide 7
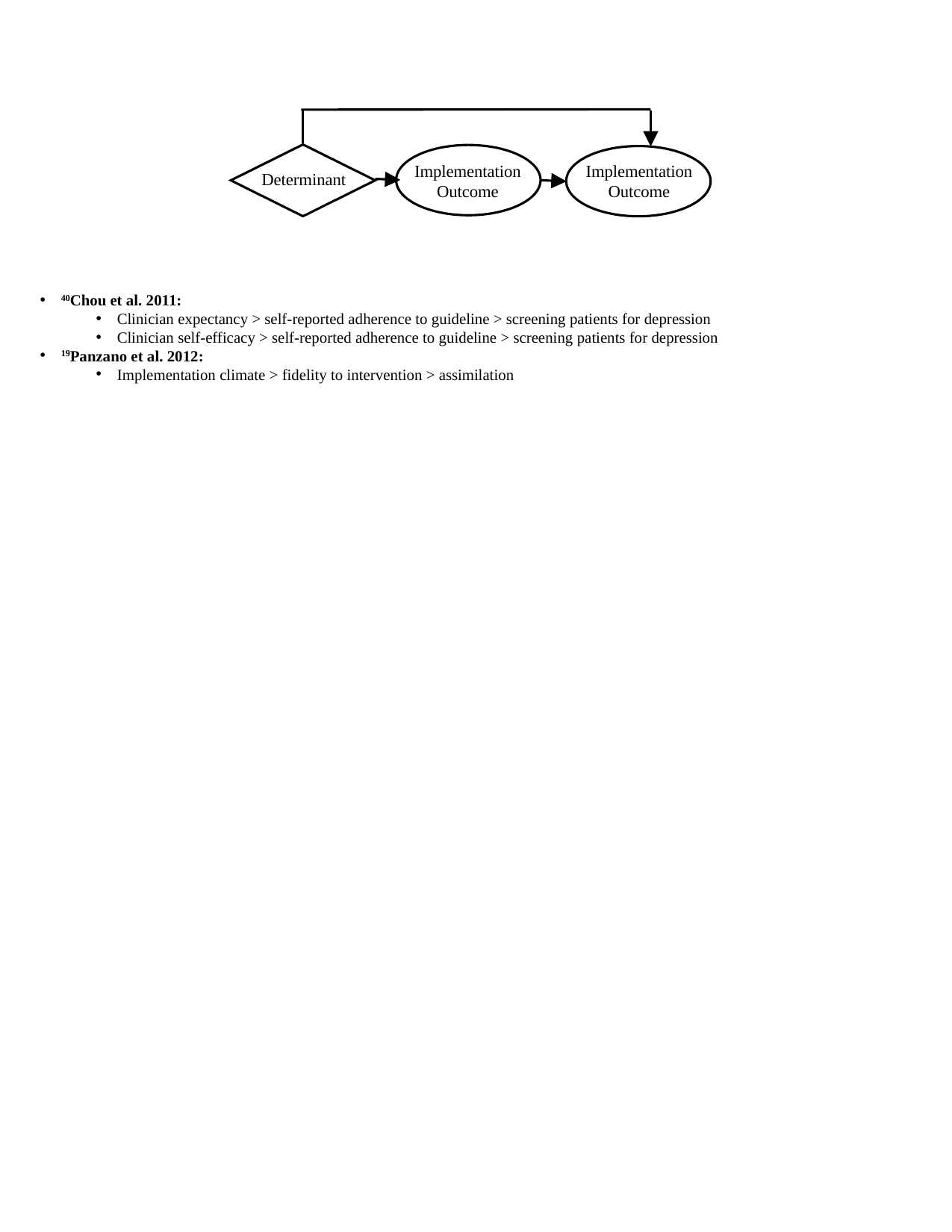

Implementation Outcome
Implementation Outcome
Determinant
40Chou et al. 2011:
Clinician expectancy > self-reported adherence to guideline > screening patients for depression
Clinician self-efficacy > self-reported adherence to guideline > screening patients for depression
19Panzano et al. 2012:
Implementation climate > fidelity to intervention > assimilation
